# A network meta-analysis of the safety of systemic treatments in patients with metastatic hormone-sensitive prostate cancer

**DOI:** 10.3389/fonc.2025.1468928

**Published:** 2025-09-22

**Authors:** Massimo Di Maio, Enrique Gonzalez-Billalabeitia, Laura Marandino, Pablo Maroto, Marc-Olivier Timsit, Irene Luccarini, Paul Robinson, Suzy Van Sanden, Boris A. Hadaschik

**Affiliations:** ^1^ Department of Oncology, University of Turin, A.O.U. Città della Salute e della Scienza di Torino, Turin, Italy; ^2^ Medical Oncology Department, Hospital Universitario 12 de Octubre, Madrid, Spain; ^3^ Department of Medical Oncology, IRCCS San Raffaele Scientific Institute, Milan, Italy; ^4^ Medical Oncology Services, Hospital de la Santa Creu i Sant Pau, Barcelona, Spain; ^5^ Departments of Urology, Hôpital européen Georges-Pompidou, Université Paris Cité, Paris, France; ^6^ EMEA Health Economics, Market Access and Reimbursement, Johnson & Johnson, Milan, Italy; ^7^ EMEA Health Economics, Market Access and Reimbursement, Johnson & Johnson, High Wycombe, United Kingdom; ^8^ EMEA Health Economics, Market Access and Reimbursement, Johnson & Johnson, Beerse, Belgium; ^9^ Department of Urology, University Hospital Essen, Essen, Germany

**Keywords:** adverse events, androgen receptor pathway inhibitors, metastatic castration-sensitive prostate cancer, metastatic hormone-sensitive prostate cancer, serious adverse events, mHSPC

## Abstract

**Background:**

The last decade saw the emergence of several new systemic therapies for metastatic hormone-sensitive prostate cancer (mHSPC). While these treatments demonstrated similar efficacy in indirect comparisons, comparisons of safety outcomes are needed to help guide the selection of treatment regimens and sequences. We conducted network meta-analyses (NMAs) comparing safety of systemic treatments for mHSPC.

**Methods:**

A systematic literature review was performed for randomized controlled trials (RCTs) investigating systemic treatments for mHSPC published before July 2022. Studies were restricted by network connectivity and study population homogeneity. Bayesian NMAs were performed for available data on grade ≥3 adverse events (AEs), serious AEs (SAEs), and any AE.

**Results:**

The study included eight RCTs (n=172–1228 by treatment arm) and seven treatment regimens: androgen deprivation therapy (ADT) alone, docetaxel plus ADT, androgen receptor pathway inhibitor (ARPI; apalutamide, enzalutamide, or abiraterone acetate plus prednisone [AAP]) plus ADT, and docetaxel plus ARPI (darolutamide or AAP) plus ADT. Apalutamide plus ADT had the lowest relative risk ([RR]; 1.18 (95% credible interval [CrI] 1.02–1.35) of grade ≥3 AEs versus ADT alone, followed by enzalutamide plus ADT (1.34 [1.17–1.52]), docetaxel plus ADT (1.44 [1.33–1.56]), AAP plus ADT (1.48 [1.39–1.58]), darolutamide plus docetaxel plus ADT (1.53 [1.33–1.72]), and AAP plus docetaxel plus ADT (1.60 [1.41–1.79]). For SAEs, RRs (95% CrI) versus ADT alone were 1.26 (1.03–1.53) for apalutamide plus ADT, 1.33 (1.12–1.57) for AAP plus ADT, 1.54 (1.28–1.84) for enzalutamide plus ADT, 3.78 (3.35–4.26) for docetaxel plus ADT, and 3.83 (3.39–4.31) for darolutamide plus docetaxel plus ADT. Similar results were observed for any AE.

**Conclusions:**

Overall, risk of grade ≥3 AEs, SAEs, and any AE was lower with doublet ARPI versus docetaxel-based doublet or triplet regimens, and apalutamide plus ADT had the lowest risk. Variability of data reporting should be considered.

## 1 Introduction

Over the last decade, androgen receptor pathway inhibitors (ARPIs), such as abiraterone acetate plus prednisone (AAP), apalutamide, enzalutamide, and darolutamide, have been investigated and approved for metastatic hormone-sensitive prostate cancer (mHSPC) as a part of doublet and/or triplet combinations with androgen deprivation therapy (ADT) (1–13). Current treatment guidelines for mHSPC recommend doublet regimens, combining ADT with an ARPI (AAP, apalutamide, or enzalutamide), or triplet regimens with ADT, an ARPI (AAP or darolutamide), and docetaxel (14, 15). Upfront external beam radiation therapy with ADT is recommended for patients with low-volume mHSPC (15). The most recent guidelines no longer recommend the docetaxel and ADT doublet regimen (14, 15). ADT alone is not recommended, except in specific cases such as for vulnerable patients for whom treatment intensification would not be tolerated (14) or asymptomatic patients with limited life expectancy or definite contraindications to the combination regimens (15).

In clinical studies, doublet regimens consisting of docetaxel (STAMPEDE and CHAARTED), AAP (STAMPEDE and LATITUDE), apalutamide (TITAN), or enzalutamide (ARCHES), all with ADT, prolonged survival versus placebo with ADT for patients with mHSPC (5, 6, 12, 16–18). Triplet regimens consisting of AAP plus docetaxel and ADT (subset of PEACE-1) and darolutamide plus docetaxel and ADT (ARASENS) have demonstrated survival benefits versus docetaxel plus ADT (11, 13, 19). No head-to-head comparisons between ARPI doublet and triplet regimens in mHSPC have been performed (14, 15).

Several indirect treatment comparisons (ITCs) have been conducted to compare triplet regimens with ARPI doublet regimens. While the available evidence was not always consistent, some studies showed no significant overall survival or progression-free survival benefit for triplet versus doublet regimens (20–24). As the primary focus of the indirect comparisons has generally been on efficacy outcomes, indirect comparisons of safety outcomes for these systemic regimens have been limited (20–26).

Comparisons based on safety outcomes are required for clear understanding of the benefit/risk ratio when considering alternative treatment regimens and sequences. We therefore conducted network meta-analyses (NMAs) comparing the safety of systemic treatment regimens for mHSPC reported in randomized controlled trials (RCTs).

## 2 Evidence acquisition

### 2.1 Study selection and search strategy

Study selection followed the systematic literature review (SLR) methodology based on the requirements from National Institute for Health and Care Excellence (NICE) (27, 28), the Preferred Reporting Items for Systematic Reviews and Meta-Analyses (PRISMA) (29), and the Guidance for Undertaking Reviews in Health Care from the University of York’s Centre for Reviews and Dissemination (30). Embase, Medline, Cochrane Central Register of Controlled Trials (CENTRAL), and Cochrane Database of Systematic Reviews (CDSR) databases were searched for RCTs published on or before July 19, 2022, in English only, via Ovid platform (Wolters Kluwer) with no restriction on the publication date. Studies from meeting proceedings and from publication citations were also included. Relevant RCTs of systemic treatments for mHSPC were identified according to the population, intervention, comparator, outcome, and study design criteria (PICOS; **Table 1**). Systemic treatments of interest were ADT alone or in combination with an ARPI, or with docetaxel, or with both docetaxel and an ARPI. The SLR search string for the final search (July 19, 2022) is shown in **Supplementary Table 1**. Two reviewers independently screened the search results at abstract and full text reviews; discrepancies were resolved by a third independent reviewer. Studies were included in the NMAs if they met all PICOS criteria and based on data availability (feasibility assessment for network connectivity and heterogeneity of reporting of adverse events). If a study did not fully meet the PICOS requirement for the M1 unrestricted population (i.e., without additional eligibility criteria) but it was required in the NMA network to allow comparison of a specific treatment, the study was considered for inclusion based on clinical expert recommendations and results were verified by sensitivity analyses (described in section 2.2.2). The search strategy allowed for identification of RCTs and non-RCTs.

**Table 1:** PICOS criteria for inclusion of safety studies.

PICOs	Inclusion	Exclusion
Patient population	• Men (aged ≥18 years) with mHSPC	• Publications reporting on patient populations in the following categories: o Females o Children o Healthy volunteers o Patients with only noncancerous prostate disease (such as benign prostatic hyperplasia) o Patients with malignancies other than prostate cancer o Patients with localized/locally advanced prostate cancer o Patients with metastatic prostate cancer who have been treated previously with hormonal therapy
Intervention and comparators	• AAP, ADT, apalutamide, darolutamide, docetaxel, enzalutamide • No restriction based on treatment comparisons reported/not reported	• Publications that do not report data specific to treatment using AAP, ADT, apalutamide, darolutamide, docetaxel, enzalutamide
Outcomes measures	• Safety o Incidence of grade ≥3 AEs o Incidence of SAEs o Incidence of AEs	• Publications that only report data on the following types of outcomes: o Pharmacokinetics/pharmacodynamics o Cost and resource use o ICERs, QALYs and other cost-effectiveness outcomes
Study design	• The review was limited to publications of studies with the following designs: o RCTs o SLRs of RCTs (only for citation review) o Prospective interventional studies	• Publications of studies with the designs outside the inclusion criteria
Restrictions	• Only English-language articles/conference abstracts were included	• Journal articles and conference abstracts without English full text

AAP, abiraterone acetate plus prednisone; ADT, androgen-deprivation therapy; AE, adverse event; ICER, incremental cost-effectiveness ratio; mHSPC, metastatic hormone-sensitive prostate cancer; QALY, quality-adjusted life year; RCT, randomized controlled trial; SAE, serious AE; SLR, systematic literature review.

Data were extracted by one researcher into a predefined Excel spreadsheet and independently checked by a second researcher. Data extraction included total population number; treatment arm; number (%) of grade ≥3 adverse events (AEs), serious AEs (SAEs), any AE, and AEs of interest; and median length of follow-up. If several data cutoffs were available for any of the included studies, the time point closest to the median follow-up of the TITAN final analysis (44 months) (6) was included to reduce heterogeneity of duration of treatment exposure (most of the included studies had a cutoff date between 40 and 50 months). Quality of studies was assessed with Cochrane Risk of Bias tool v1 (31) by one reviewer and verified by a second reviewer.

### 2.2 Statistical analyses

#### 2.2.1 NMAs

NMAs were performed for the aggregated safety outcomes: grade ≥3 AEs, SAEs, and any treatment-emergent AEs, as well as AEs of interest from included studies. AEs of interest were selected based on the well-known associations between AEs and different treatments as well as on availability of consistent data across studies. For example, rash and hypertension are known AEs of interest for apalutamide and AAP, respectively (6, 11, 12, 16, 32). Cognitive impairment, memory loss, and fatigue were associated with enzalutamide (5, 33, 34). Neutropenia, and fatigue were associated with docetaxel-based regimens (11, 13, 17, 18, 35).

Both fixed-effects and random-effects Bayesian NMAs were conducted according to the NICE-recommended methods (36, 37). The key assumptions for NMA were that underlying relative treatment effects were the same in all trials, treatment effect modifiers had only absolute effects, and common comparators used to link treatments were identical, e.g., small differences in dose or schedule did not affect relative effects. For each safety outcome, an estimate of the relative effect of interest was reported as relative risk (RR) with a 95% credible interval (CrI), displayed in a matrix for all possible treatment regimen comparisons and in a forest plot versus the comparator ADT alone. Systemic treatment regimens were ranked according to the surface under the cumulative ranking curve (SUCRA). All Bayesian analyses were conducted in WinBUGS 1.4.3 (MRC Biostats Unit at Cambridge) using three chains each with 50,000 iterations for “burn-in” and 50,000 iterations for the posterior. A network plot showing connectivity for safety outcomes was included with the studies for each link listed. Inconsistency was assessed with a chi-square test.

#### 2.2.2 Sensitivity analyses for NMAs

Sensitivity analyses were conducted to assess assumptions and impact of study heterogeneity regarding inclusions and exclusions. For each sensitivity analysis, an NMA was performed for the safety outcomes: the aggregated treatment-emergent AEs (grade ≥3 AEs, SAEs, and any AE) and AEs of interest (results not shown).

## 3 Evidence synthesis

### 3.1 Study inclusion and study characteristics

**Figure 1:**
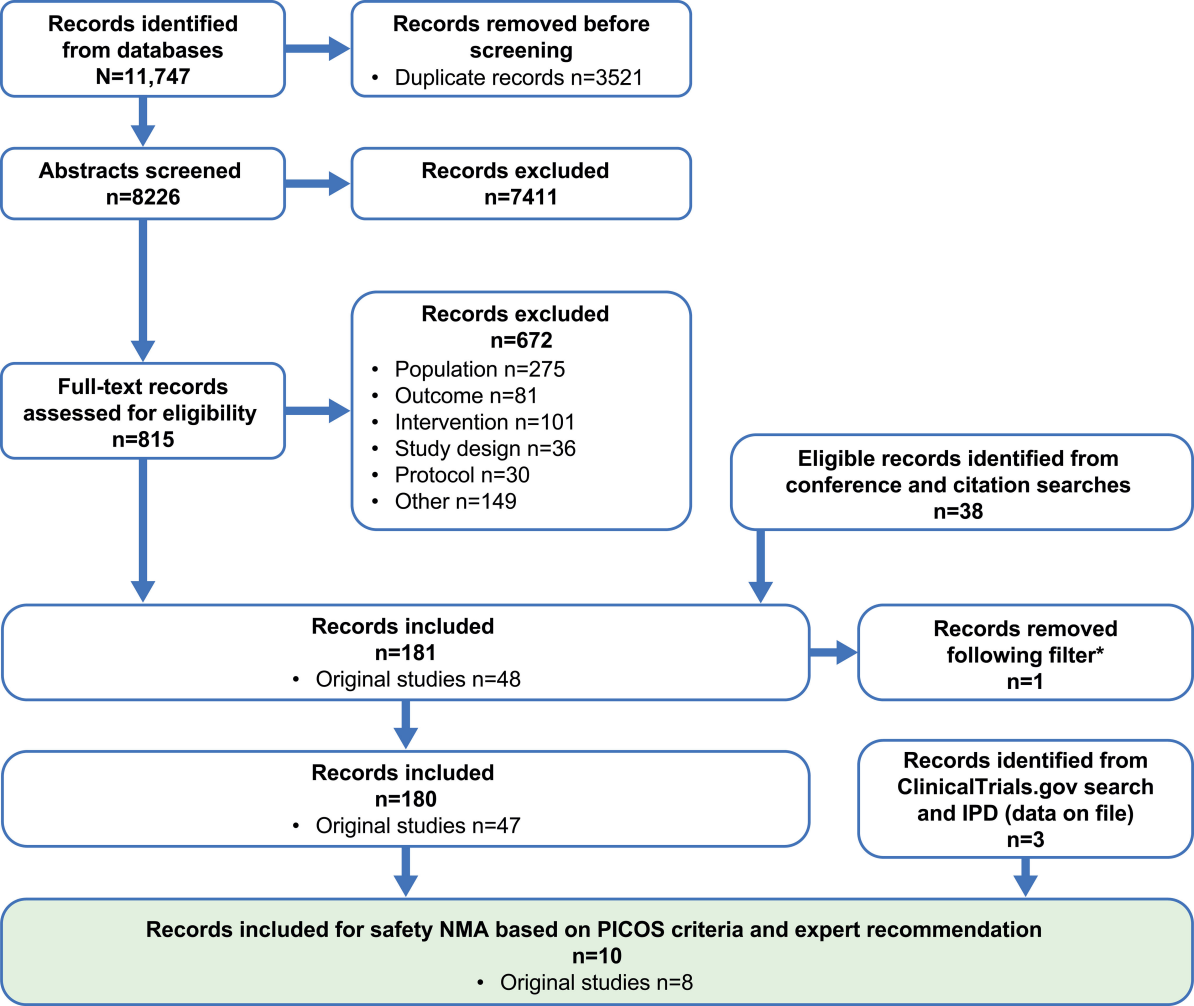
PRISMA flow diagram. Searches were conducted on several dates and combined; the final search was conducted on July 19, 2022. *A filter was applied to the original systematic literature review; one study that only reported subgroup data was removed. NMA, network meta-analysis; PICOS, population, intervention, comparator, outcome, and study design.

**Figure 2:**
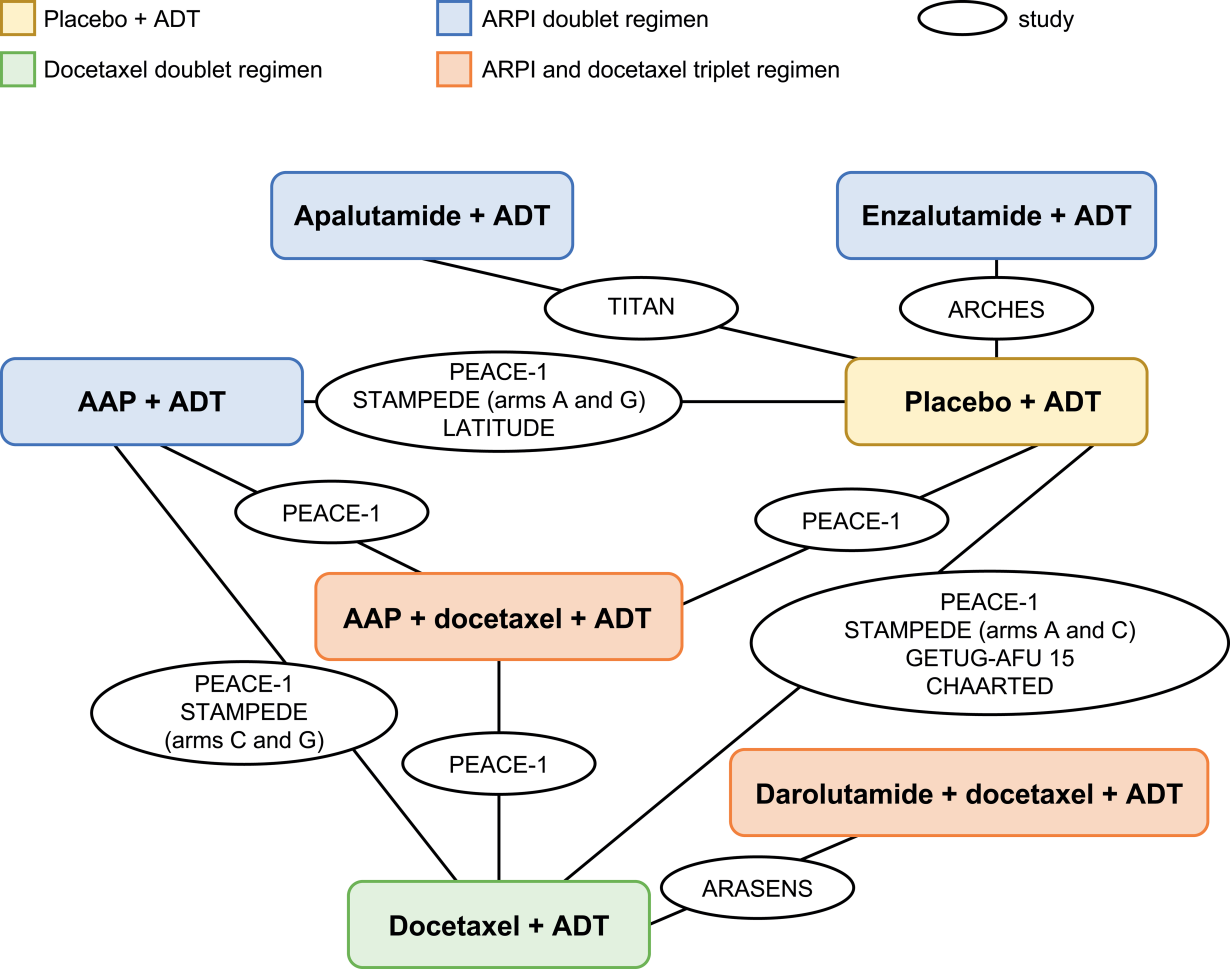
Base-case network plot. AAP, abiraterone acetate plus prednisone; ADT, androgen-deprivation therapy; ARPI, androgen receptor pathway inhibitor.

The Embase, Medline, CENTRAL, and CDSR database search identified 11,747 records; 39 additional eligible records were identified from conferences, citation searches, and ClinicalTrials.gov (**Figure 1**). Of the identified records, only eight phase 3 RCTs were included for the safety NMAs, and seven met the prespecified PICOS criteria (ARASENS, ARCHES, CHAARTED, GETUG-AFU 15, PEACE-1, STAMPEDE, and TITAN). One additional phase 3 RCT (LATITUDE) was added based on clinical expert recommendations given that it is a large study of AAP providing safety data in an mHSPC population (**Figure 2**, **Table 2**). The risk of bias in the RCTs was low, consistent with the previous report (23).

**Table 2:** Patient characteristics from the eight included studies (efficacy populations).

Study source	Disease state	Treatment groups	Number of patients	High-volume vs. low-volume,* n (%)	Synchronous vs. metachronous,^†^ n (%)	M0 vs. M1,^‡^ n (%)	Prior docetaxel, n (%)
STAMPEDE (arms G and A) James, 2017 (16)	HSPC	AAP + ADT	960	NA	465 (48) vs. 35 (4)	460 (48) vs. 500 (52)	Not included
		Placebo + ADT	957		476 (50) vs. 26 (3)	455 (48) vs. 502 (52)	
STAMPEDE (arms G and C) Sydes M, 2018 (38)	HSPC	AAP + ADT	377	NA	NA	150 (40) vs. 227 (60)	Not included
		Docetaxel + ADT	189		NA	74 (39) vs. 115 (61)	
STAMPEDE (arms C and A) James, 2016 (17) Clark, 2020 (40)	HSPC	Docetaxel + ADT	592	148 (29) vs. 354 (71)^§^	347 (59) vs. 15 (3)	230 (39) vs. 362 (61)	Not included
		Placebo + ADT	1184	320 (31) vs. 698 (69)^§^	690 (58) vs. 34 (3)	460 (39) vs. 724 (61)	
LATITUDE Fizazi, 2017 (41) Fizazi, 2019 (12)	High-risk mHSPC^║^	AAP + ADT	597	487 (82) vs. 110 (18)	597 (100) vs. 0	0 vs. 597 (100)	Not included
		Placebo + ADT	602	468 (78) vs. 133 (22)	602 (100) vs. 0	0 vs. 602 (100)	
CHAARTED Sweeney 2015 (18) Kyriakopoulos, 2018 (39)	mHSPC	Docetaxel + ADT	397	263 (66) vs. 134 (34)	289 (73) vs. 108 (27)	0 vs. 397 (100)	Not included
		Placebo + ADT	393	250 (64) vs. 143 (36)	286 (73) vs. 106 (27)	0 vs. 393 (100)	
ARASENS Smith 2022 (13) Hussain 2023 (19)	mHSPC	Darolutamide + docetaxel + ADT	651	497 (76) vs. 154 (24)	558 (86) vs. 86 (13)	0 vs. 651 (100)	Not included
		Docetaxel + ADT	654	508 (78) vs. 146 (22)	566 (87) vs. 82 (13)	0 vs. 654 (100)	
ARCHES Armstrong, 2022 (5)	mHSPC	Enzalutamide + ADT	574	354 (62) vs. 220 (38)	402 (70) vs. 83 (14)	NA vs. 536 (93)^¶^	103 (18)
		Placebo + ADT	576	373 (65) vs. 203 (35)	365 (63) vs. 86 (15)	NA vs. 531 (92)^¶^	102 (18)
GETUG AFU 15 Gravis, 2013 (35) Gravis, 2018 (42)	mHSPC	Docetaxel + ADT	192	92 (48) vs. 100 (52)	128 (67) vs. 62 (32)	NA vs. 190 (99)	Not included
		Placebo + ADT	193	91 (47) vs. 102 (53)	144 (75) vs. 46 (24)	NA vs. 190 (98)	
TITAN Chi, 2019 (32)	mHSPC	APA + ADT	525	325 (62) vs. 200 (38)	411 (78) vs. 85 (16)	0 vs. 525 (100)	58 (11)
		Placebo + ADT	527	335 (64) vs. 192 (36)	441 (84) vs. 59 (11)	0 vs. 527 (100)	55 (10)
PEACE-1 Fizazi, 2022 (11)	mHSPC	AAP + docetaxel + ADT (± RT)	355	224 (63) vs. 131 (37)	355 (100) vs. 0	0 vs. 355 (100)	Not included
		Docetaxel + ADT (± RT)	355	232 (65) vs. 123 (35)	355 (100) vs. 0	0 vs. 355 (100)	
		AAP + ADT (± RT)	583	331 (57) vs. 252 (43)	583 (100) vs. 0	0 vs. 583 (100)	
		Placebo + ADT (± RT)	589	336 (57) vs. 253 (43)	589 (100) vs. 0	0 vs. 589 (100)	

AAP, abiraterone acetate plus prednisone; ADT, androgen deprivation therapy; NA, not available.

*High-volume is defined in all studies, except for STAMPEDE (arms G and A and arms G and C comparisons) and TITAN, by CHAARTED criteria: visceral metastasis, or ≥4 bone lesions with ≥1 in a bony structure beyond vertebral bodies and pelvis. Any disease not meeting high-volume criteria is classified as low-volume. High-volume in TITAN is defined by modified CHAARTED criteria: visceral metastases and ≥1 bone lesion, or ≥4 bone lesions with ≥1 outside the axial skeleton. Low-volume disease in TITAN is defined as bone lesions not meeting the definition of high-volume disease. †Synchronous disease is defined as metastases at initial diagnosis; metachronous disease is defined as metastases developed after localized disease. ^‡^M0 – no distant metastases, M1 – distant metastases. ^§^Percentages are calculated using the denominators that include patients with metastatic disease at baseline whose metastatic burden was assessed from scans and patients with nonmetastatic disease at baseline: n = 502 for docetaxel + ADT and n=1018 for placebo + ADT. ^║^Defined as ≥2 of 3 high-risk factors (1): a Gleason score ≥8, ≥3 bone lesions, and measurable visceral metastasis. ^¶^Confirmed metastases as assessed by independent central review after investigator assessment at study entry.

Systemic treatment regimens from the eight RCTs with median follow-up times between 29 and 53 months were analyzed according to aggregated safety outcomes [grade ≥3 AEs, SAEs, and any AE (**Table 3**, **Figure 2**)].

**Table 3:** Adverse events reported from eight included studies (safety populations).

Study source	Treatment groups	Number of patients	Grade ≥3 AEs n (%)	SAEs n (%)	Any AE n (%)	Follow-up mo (IQR)
STAMPEDE (arms G and A) James, 2017 (16)	AAP + ADT	948	443 (47)*	NA	943 (99)*	40
	Placebo + ADT	960	315 (33)*	NA	950 (99)*	
STAMPEDE (arms G and C) Sydes M, 2018 (38)	AAP + ADT	373	180 (48)*	NA	370 (99)*^,†^	48
	Docetaxel + ADT	172	86 (50)*	NA	172 (100)*^,†^	
STAMPEDE (arms C and A) James, 2016 (17)	Docetaxel + ADT	550	288 (52)*	NA	550 (100)*^,†^	43 (30-60)
	Placebo + ADT	1228	399 (32)*	NA	1213 (99)*^,†^	
LATITUDE Generated from IPD; data on file; Fizazi, 2019 (12)	AAP + ADT	597	411 (69)	210 (97)	572 (96)	51.8 (47.2−57.0)
	Placebo + ADT	602	309 (51)	162 (27)	566 (94)	
CHAARTED Clinicaltrials.gov (43)	Docetaxel + ADT	390	NA	116 (30)	NA	29
	Placebo + ADT	392	NA	12 (3)	NA	
ARASENS Smith, 2022 (13)	Darolutamide + docetaxel + ADT	652	458 (70)^‡^	292 (45)^‡^	649 (100)^‡^	43.7
	Docetaxel + ADT	650	439 (68)^‡^	275 (42)^‡^	643 (99)^‡^	42.4
ARCHES Armstrong, 2022 (5)	Enzalutamide + ADT	572	224 (39)^§^	197 (34)	520 (91)	44.6
	Placebo + ADT	574	160 (28)^§^	128 (22)	504 (88)	
GETUG AFU 15 Gravis G, 2013 (35)	Docetaxel + ADT	189	NA	72 (38)^†^	NA	50 (39–63)
	Placebo + ADT	186	NA	0 (0)^†^	NA	
TITAN Generated from IPD; data on file	APA + ADT	524	262 (50)	156 (30)	510 (97)	44
	Placebo + ADT	527	228 (43)	125 (24)	512 (97)	
PEACE-1 Fizazi, 2022 (11)	AAP + docetaxel + ADT (± RT)	347	217 (63)	NA^║^	346 (100)^¶^	52.8**
	Docetaxel + ADT (± RT)	350	181 (52)	NA^║^	349 (100)^¶^	
	AAP + ADT (± RT)	226	149 (66)	NA^║^	226 (100)^¶^	
	Placebo + ADT (± RT)	237	97 (41)	NA^║^	233 (98)^¶^	

AAP, abiraterone acetate plus prednisone; ADT, androgen deprivation therapy; AE, adverse event; IPD, individual participant data; NA, not available; RT, radiotherapy; SAE, serious adverse event.

*Safety data for M1 + M0 STAMPEDE population were reported together. ^†^A continuity correction was performed where n=0.5 was added to each AE when performing the network meta-analyses. ^‡^Reported AEs are for each grade (worst only) and summed together. ^§^Grade 3–4 AEs, instead of grade 3–5, were included based on availability. ^║^SAEs were defined as grades 3–5, which was not consistent with other trial definitions of SAEs and therefore the data were not included. ^¶^A continuity correction was performed where n=1 was added to each AE when performing the network meta-analyses. **Follow-up for OS in the overall population.

The seven systemic treatment regimens included were ADT alone, three ARPI doublet regimens (ADT with either AAP, apalutamide, or enzalutamide), docetaxel with ADT, and two triplet regimens (ADT with either AAP plus docetaxel or darolutamide plus docetaxel).

ENZAMET (34), comparing ADT plus enzalutamide versus ADT plus a first-generation nonsteroidal antiandrogen (NSAA), was excluded because patients were permitted to receive concomitant docetaxel (and safety data were not reported separately); moreover, the study would only connect to the network via its enzalutamide plus ADT treatment arm, thereby only providing additional comparisons versus ADT plus NSAA, which are of limited clinical relevance. The effect of the inclusion of ENZAMET in NMA was explored in sensitivity analyses.

There were some differences in patient population, study treatment, and reporting of safety outcomes among the included RCTs (**Tables 2**, **3**). STAMPEDE included patients with either nonmetastatic (M0) or metastatic (M1) disease (16, 17, 38) and PEACE-1 and LATITUDE included only patients with synchronous (*de novo*) and synchronous high-risk M1 disease, respectively (11, 12). Other RCTs, such as CHAARTED, ARASENS, ARCHES, and TITAN, had a high proportion of patients with synchronous and high-volume disease (5, 13, 18, 19, 32, 39). Patients in PEACE-1 received systemic treatments with or without radiotherapy (11) and some patients (11% and 18%) from TITAN and ARCHES had received prior docetaxel treatment before the start of the study (32, 33). ARCHES investigators reported grade 3–4 AEs instead of grade ≥3 AEs (5). The definition of ADT (surgical and/or medical castration) and the duration of docetaxel administration varied between studies. Patients with history of seizure were excluded from TITAN (32) and from ARCHES (33) but not from ARASENS (13). Inclusion of these RCTs with slightly different patient populations was considered acceptable as these variations were not expected to substantially bias the analysis.

### 3.2 NMAs

The overall base-case network plot with all eight RCTs (range for treatment arms, n=172–1228) is shown in **Figure 2**. The network plots for grade ≥3 AEs and any AE or for SAEs are shown in **Supplementary Figure 1**.

#### 3.2.1 NMAs of grade ≥3 AEs, SAEs, and any AE

**Figure 3:**
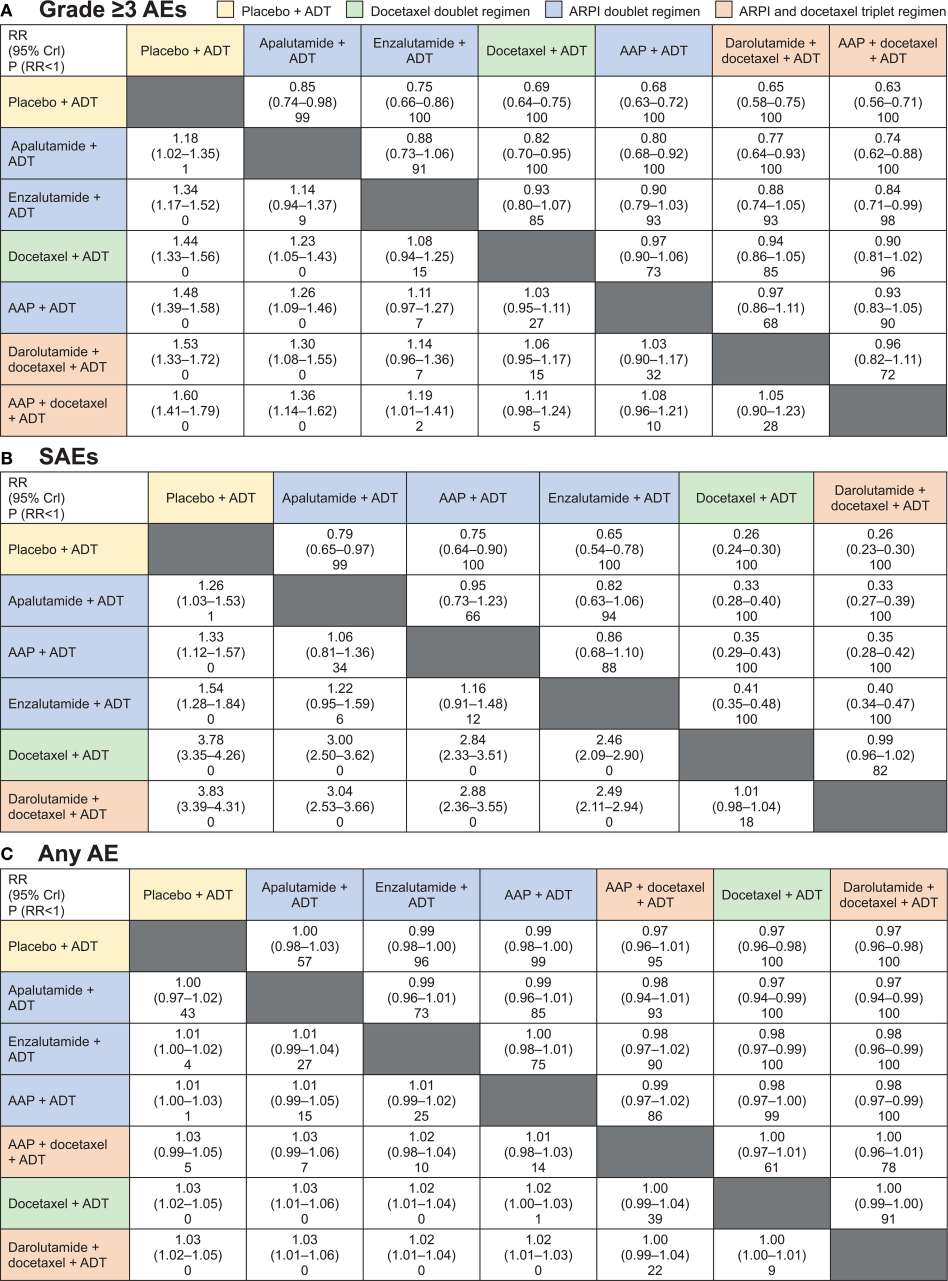
Fixed-effects model for Bayesian network meta-analysis comparison results for **(A)** grade ≥3 adverse events (AEs), **(B)** SAEs, and **(C)** any AE. Treatment regimens are ordered according to surface under the cumulative ranking curve. AAP, abiraterone acetate plus prednisone; ADT, androgen-deprivation therapy; ARPI, androgen receptor pathway inhibitor; CrI, credible interval; P, probability; RR, relative risk. Cells contain RR (95% Crl) and P (RR<1) row versus column.

The grade ≥3 AEs, SAEs, and any AE from each trial are shown in **Table 3**. The fixed-effects model was selected as the base-case for all outcomes shown in this article over the random-effects model shown in the supplement as most networks were small with little repetition of studies, minimizing the need to estimate the between-study variability. All treatment regimens presented in the overall network plot were included in the grade ≥3 AEs and any AE NMAs, except for CHAARTED and GETUG-AFU 15, for which data were not available. Data for SAEs from PEACE-1 and STAMPEDE were also not available. According to SUCRA ranking, ARPI doublet regimens consistently ranked above the docetaxel doublet and triplet regimens for grade ≥3 AEs, SAEs, and any AE in most cases [all except AAP plus ADT for grade ≥3 AEs (**Figure 3**; see **Supplementary Figure 2** for random-effect model)].

**Figure 4:**
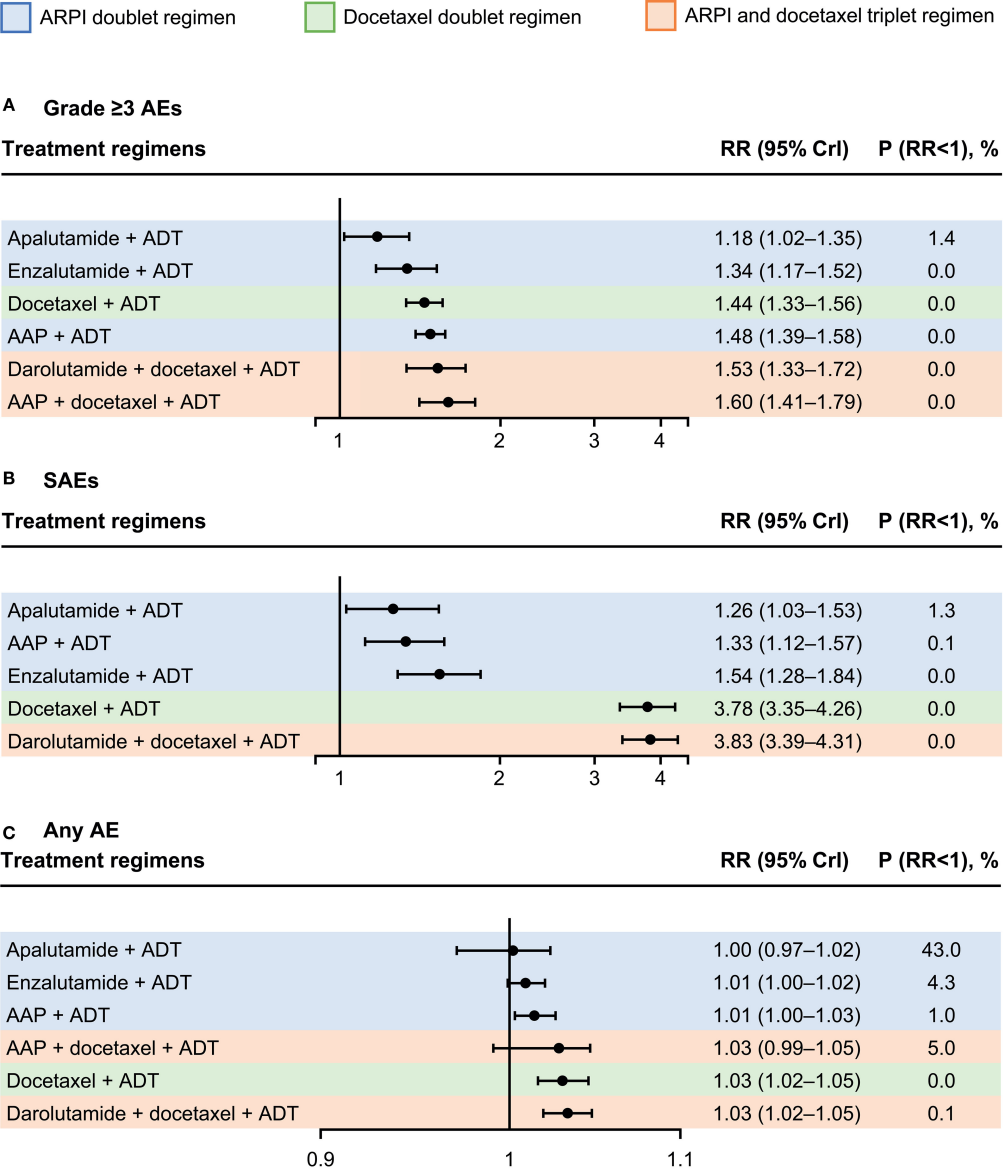
Relative risk (RR) for aggregated safety outcomes following systemic therapies versus androgen-deprivation therapy (ADT) alone. **(A)** grade ≥3 adverse events (AEs), **(B)** SAEs, and **(C)** any AE. All data are rounded from fixed-effects model. Treatment regimens are ordered according to surface under the cumulative ranking curve. AAP, abiraterone acetate plus prednisone; ARPI, androgen receptor pathway inhibitor; CrI, credible interval.

Forest plots comparing doublet and triplet interventions with ADT alone for each aggregated AE outcome are shown in **Figure 4**. For grade ≥3 AEs, apalutamide had the lowest RR [95% credible interval (CrI)] [1.18 (1.02–1.35)], followed by enzalutamide [1.34 (1.17–1.52)], docetaxel [1.44 (1.33–1.56)], and AAP [1.48 (1.39–1.58)], then the triplet regimens, darolutamide with docetaxel [1.53 (1.33–1.72)] and AAP with docetaxel [1.60 (1.41–1.79)], all with ADT. For SAEs, the RRs with ARPI-based doublet regimens versus ADT alone were considerably lower per non-overlapping 95% CrIs than those with the docetaxel-based doublet and triplet regimens versus ADT alone. RR for SAEs was lowest with apalutamide [1.26 (1.03–1.53)], followed by AAP [1.33 (1.12–1.57)], enzalutamide [1.54 (1.28–1.84)], and docetaxel [3.78 (3.35–4.26)], and then the triplet regimen darolutamide plus docetaxel [3.83 (3.39–4.31)], all with ADT.

The analyses for any AE were more difficult as almost all patients reported an AE. This resulted in a boundary issue where all the RRs were close to 1 (**Figure 4C**) and inability to differentiate between the treatment regimens. Despite this limitation, RR point estimates were lower for ARPI doublets than for docetaxel-based regimens.

The only inconsistency between the direct and indirect evidence was found for grade ≥3 AEs for AAP plus ADT (STAMPEDE arms G and C) versus docetaxel plus ADT (PEACE-1) driven by PEACE-1 data. The inconsistency was addressed in the sensitivity analysis described below.

Forest plots based on the random-effects model showed similar findings (**Supplementary Figure 3**).

#### 3.2.2 NMA of AEs of interest

AEs of interest for each RCT are summarized in **Supplementary Table 2**. Not all treatments could be compared for each AE of interest due to availability and comparability of the data. Data for fatigue, neutropenia, hypertension, rash, fall, and cognitive impairment were considered suitable for analysis. Data were extracted for cardiovascular disease and fractures; however, due to heterogeneity of reporting, an NMA could not be conducted.

Forest plots comparing interventions with ADT alone for each AE of interest (grades ≥3 and any grade) are shown in **Supplementary Figure 4**. Comparisons between ARPI, docetaxel doublet, and docetaxel triplet regimens were conducted for fatigue, neutropenia, and hypertension; due to data availability, only ARPI doublet regimens were included in the analyses for hypertension (any grade), rash, fall, and cognitive impairment.

For grade ≥3 AEs, versus ADT alone, ARPI doublet regimens had lower RRs for fatigue and neutropenia than docetaxel-based doublet and triplet regimens. For hypertension, docetaxel doublet regimen had the lowest RR, followed by apalutamide, the docetaxel triplet regimens, and then enzalutamide and AAP doublet regimens, all with ADT. Among ARPI doublet regimens, apalutamide had the lowest RR for grade ≥3 fatigue, hypertension, and fall and the highest for neutropenia and rash; AAP had the lowest RR for neutropenia, rash, and cognitive impairment and the highest for hypertension. Enzalutamide had the highest RR for grade ≥3 fatigue, fall, and cognitive impairment. None of the RRs for enzalutamide were among the lowest.

For any-grade AEs, versus ADT alone, ARPI doublet regimens also had lower RRs for fatigue and neutropenia than docetaxel-based doublet and triplet regimens. Among ARPI doublet regimens, apalutamide had the lowest RR for any-AE neutropenia, hypertension, and fall and the highest for rash and cognitive impairment; AAP had the lowest RR for fatigue, rash, and cognitive impairment and the highest for hypertension. Enzalutamide had only the highest RR for any-AE fatigue, neutropenia, and falls.

### 3.3 Sensitivity analyses

In a sensitivity analysis, ENZAMET was connected to the network under the assumption that the treatment regimen, ADT plus a first-generation NSAA, was equivalent to ADT. Results remained consistent with the base-case with the inclusion of ENZAMET (**Supplementary Figure 5**).

Sensitivity analyses that excluded, separately, STAMPEDE (M0 and M1 population), LATITUDE (synchronous high-risk M1 population), PEACE-1 (patients received study treatment ± radiotherapy), and ARCHES (reported grade 3–4 AEs instead of grade ≥3 AEs) were also conducted (**Supplementary Figures 6**–**9**, respectively). To check the impact of prior treatment with docetaxel, a sensitivity analysis was conducted that excluded patients from the TITAN study who received prior docetaxel (**Supplementary Figure 10**). For all sensitivity analyses conducted, results remained generally consistent with the base-case analysis.

Another sensitivity analysis was performed that included only licensed treatment regimens for mHSPC in Europe by European Medicines Agency/The Medicines and Healthcare products Regulatory Agency. Therefore, this analysis included data only from TITAN, ARCHES, LATITUDE, STAMPEDE arms A and C, and ARASENS (**Supplementary Figure 11**) (7–10). PEACE-1 was not included. This sensitivity analysis was performed for grade ≥3 AEs and any AE only. Because the data for SAEs from STAMPEDE were not reported, the sensitivity analysis for this endpoint would encompass the exact same trials as in the base-case analysis (**Figure 4B**). Similar to the base-case analysis for grade ≥3 AEs, apalutamide plus ADT compared with ADT alone had the lowest RR (95% CrI) [1.18 (1.02–1.36)], followed by enzalutamide plus ADT [1.35 (1.17–1.55)], AAP plus ADT [1.51 (1.34–1.69)], docetaxel plus ADT [1.57 (1.43–1.73)], and darolutamide plus docetaxel plus ADT [1.66 (1.44–1.88)]. The RRs for any AE compared with ADT alone were also consistent with the base-case analysis: 1.00 (0.95–1.04) for apalutamide, 1.02 (1.00–1.04) for enzalutamide, 1.02 (0.99–1.04) for AAP, 1.06 (1.03–1.08) for docetaxel, and 1.06 (1.04–1.08) for darolutamide plus docetaxel, all with ADT. Exclusion of PEACE-1 from this analysis did not impact the results, suggesting that the inconsistency between STAMPEDE arms G and C and PEACE-1 is minor.

## 4 Discussion

Where there is a clear differentiation between treatment options based on efficacy outcomes, without notable differences in safety and quality of life, it is reasonable to assume that patients and clinicians are likely to choose the most effective treatment. However, in the mHSPC disease setting, numerous available treatment regimens with similar efficacy outcomes, compared with ADT alone, are recommended by current treatment guidelines, including ARPI-based doublet regimens and docetaxel-ARPI–based triplet regimens (14, 15, 44). The 2024 Advanced Prostate Cancer Consensus Conference (APCCC), consisting of 120 international prostate cancer experts, did not reach consensus on whether triplet regimens should be the preferred choice for high-burden mHSPC: 54% of experts would recommend triplet therapies for most patients, while 40% would recommend them to only selected patients (44). APCCC acknowledged that it is still unknown which patients will benefit the most from triplet regimens and whether triplet regimens are superior to doublet regimens.

In situations in which treatment options cannot be meaningfully differentiated by efficacy outcomes or clinical recommendations, differences in safety may carry additional importance and help guide treatment decisions. Patients and their clinicians may consider the risk and potential severity of all AEs as well as specific types of AEs that they personally wish to avoid when deciding on their treatment plan. Knowledge of treatment toxicity would also be useful for assessing the benefit/risk ratios of treatments with different efficacy. This NMA study aimed to provide indirect comparisons of safety outcomes of systemic treatments for mHSPC where direct head-to-head studies are unavailable. Our study fills an evidence gap for clinicians and patients who are developing informed treatment plans.

The results of our NMAs of aggregated AEs showed a consistent and clinically meaningful reduced risk of grade ≥3 AEs, SAEs, and any AE with ARPI-doublet regimens versus the docetaxel-based doublet and triplet regimens. Doublet treatment with an ARPI plus ADT was associated with an overall lower risk of the aggregated any AE, grade ≥3 AEs, and SAEs compared with ADT alone, than triplet regimens. The risk of SAEs with ARPI-based doublet regimens over the docetaxel-based doublet and triplet regimens was considerably lower based on clearly separated 95% CrIs. The doublet regimen of apalutamide plus ADT was associated with the lowest risk among all doublet combination treatments for grade ≥3 AEs, SAEs, and any AE despite the assessed populations having unfavorable prognostic characteristics, such as synchronous disease. Therefore, apalutamide demonstrated a favorable safety profile compared with all alternative doublet treatments.

In our study, different treatment regimens had varying levels of risk for each AE of interest. It is important to note that, due to limitations associated with data availability and inconsistency of reporting across RCTs, the number and type of treatment regimens included in the NMAs for each comparison differed. For example, for rash and fall, only a comparison between ARPI doublet regimens was possible. For fatigue (any grade and grade ≥3) and for falls (any grade), a higher risk was observed with enzalutamide plus ADT than with other ARPIs. Apalutamide plus ADT showed a higher risk for rash than the other doublet regimens. There was higher risk of fatigue (any grade and grade ≥3) with docetaxel-based regimens versus any ARPI doublet regimen. ARPI doublet regimens showed a lower risk for neutropenia. The risk of specific AEs, in addition to aggregated AEs, should be included in the discussion with the patient during treatment selection.

ARPIs are generally well tolerated, and have manageable safety profiles. As docetaxel is known to be associated with more severe side effects, such as neutropenia (38), compared with ARPIs, it is generally only recommended for treatment of patients considered “fit” for chemotherapy; however, the criteria for fitness for docetaxel treatment remain undefined (44). Current guidelines do not recommend docetaxel doublet regimen and, when a triplet regimen is considered, the European Association of Urology guidelines recommend ensuring patients understand that docetaxel is the driver of the side effects reported for triplet regimens (14, 15). The results of our study, showing that the inclusion of docetaxel in ARPI-triplet regimens leads to important additional tolerability considerations compared with ARPI-doublet regimens, align with these recommendations.

Furthermore, our results were generally consistent with those of previously conducted NMAs for safety outcomes; however, these ITCs had more limited scopes than our study (26, 45, 46). In addition, these studies were conducted with different ITC methodologies and/or assumptions. The differences included the format of safety data used (45), the lack of ARPI plus ADT doublet regimens (26), and lack of further comparisons beyond ARPI doublet regimens (46). Furthermore, the focus of these ITCs was more on efficacy than on safety as the safety analyses were generally less detailed (26, 45, 46). The variability in the published ITCs conducted for safety results and incomplete list of included studies prevent direct comparison of our study with them.

Our NMA has some limitations, principally the necessary use of aggregated data from the majority of included RCTs that could be a source of potential confounders. We checked definitions and reporting criteria in the included RCTs and found them similar for most of the safety outcome data, although some networks included fewer interventions for comparisons of the AEs of interest. However, during the feasibility assessment, general consistency of the selected aggregated safety outcomes was established across included RCTs. This assessment allowed us to be reasonably confident that the safety outcomes for missing AEs of interest would be consistent with our results.

Differences in RCT populations, safety reporting, and treatment exposure were another limitation. Some populations either consisted entirely of patients with synchronous mHSPC or included a large proportion of such patients. Additionally, some RCTs included large proportions of patients with high-volume disease. STAMPEDE included patients with both metastatic and non-metastatic disease, LATITUDE included synchronous high-risk patients, TITAN and ARCHES included a proportion of patients who received prior docetaxel, and PEACE-1 included only patients with synchronous metastases. However, considering that the endpoint of our NMA study was safety and not efficacy, we preferred to include these large, broad populations despite heterogeneity of some characteristics. Sensitivity analyses excluding all patients from LATITUDE, ARCHES, or STAMPEDE, or those who received prior docetaxel in TITAN, had only a minor impact on the results. Due to the inaccessibility of patient-level data, we were unable to exclude patients with prior docetaxel from ARCHES. However, based on the TITAN sensitivity analysis results, it is reasonable to infer that excluding these patients from ARCHES would likely have a similarly minor impact. The safety outcomes of PEACE-1 were reported by intervention with or without radiotherapy. However, according to our sensitivity analysis without PEACE-1, the enrollment of patients with synchronous disease or inclusion of radiotherapy did not affect the results. Therefore, these inconsistencies did not greatly influence our findings. Shorter exposure to treatment can be attributed to high-risk features of patient populations in some RCTs; however, the risk of AEs in these studies may be compensated for by the longer exposure and cumulative toxicity in the other studies. Moreover, we included the data, where available, with the follow-up closest to that of TITAN, thereby reducing heterogeneity of treatment exposure as far as possible. Differences in safety outcomes observed in our study were best represented by the grade ≥3 AE and SAE results. Differentiating between RRs for any AE was difficult because of the boundary issue. Nevertheless, the results for any AEs were generally consistent with those for grade ≥3 and SAEs.

Another limitation is the possibility that further RCTs have been reported since the final literature search date on July 19, 2022. Of note, the ARANOTE study, which assessed darolutamide plus ADT versus ADT alone for treatment of mHSPC was published too late to be included in our study. While descriptive comparisons of AE rates across studies should be done with caution due to possible inconsistency in safety reporting between studies, the relative safety results from ARANOTE showed similar rates of grade 3–4 and serious AEs between darolutamide plus ADT and placebo plus ADT (31% vs. 30% and 24% vs. 24%, respectively) (47). As such it is unlikely that ARANOTE would significantly impact the overall conclusions of our study relating to the safety profiles of doublet ARPI versus triplet ARPI regimens.

Further clinical research in mHSPC is active, with multiple studies exploring novel therapeutic targets, such as radiotherapeutics, or those in more specific populations, such as PTEN-deficient mHSPC, and patients with mutations in homologous recombination repair genes (48–51). Future NMAs will need to carefully consider the increased heterogeneity of patient populations enrolled in these ongoing studies, as previously demonstrated in mCRPC (52).

Despite the known limitations, our NMAs, with the support of the sensitivity analyses, suggest that the methodology, observed results, and conclusions are robust. Only recent, high-quality phase 3 RCTs were included, and transparent detailed descriptions of our search strategy, data extraction process, and analytical methods, as well as strict adherence to PRISMA and NICE guidelines should provide confidence in the reported results.

Our findings have potential clinical implications for treatment selection in mHSPC. Patients with comorbid conditions, such as hypertension and fatigue, need to be carefully monitored. Fall-prevention programs should be in place for older patients with mHSPC. Treatment tolerability and individual AEs should be considered and addressed according to age and physiological status. Treatment selection should be based on informed treatment decisions and patient preferences.

## 5 Conclusions

Compared with ADT alone, the risk of aggregated grade ≥3 AEs, SAEs, and any AE was consistently lower with doublet ARPI regimens than with docetaxel-based doublet or triplet regimens in patients with mHSPC in our NMA. Despite being associated with the highest risk of rash, apalutamide plus ADT demonstrated an overall favorable safety profile compared with other doublet and triplet regimens. The risk of specific AEs of interest, such as hypertension, fall, and cognitive impairment, varied among the different ARPIs. Given the lack of clinical consensus on the use of triplet treatments and the similar efficacy among the recommended systemic treatment regimens, the results of this safety NMA study can contribute to informed treatment decisions for patients with mHSPC.

## Data Availability

The data analyzed in this study is subject to the following licenses/restrictions: The data sharing policy of Janssen Pharmaceutical Companies of Johnson & Johnson is available at https://www.janssen.com/clinical-trials/transparency. Requests for access to the TITAN study data can be submitted through Yale Open Data Access (YODA) Project site at http://yoda.yale.edu. Data from the other studies used as sources for the NMA are not available from Janssen, but they are publicly available in the published literature. The other studies are not sponsored by Janssen and their data sets are publicly available by the respective study sponsors.
