# Supplementary material for: A network meta-analysis of the safety of systemic treatments in patients with metastatic hormone-sensitive prostate cancer
Source: Front Oncol. 2025 Sep 22;15:1468928. doi: 10.3389/fonc.2025.1468928 (PMC12498157; doi:10.3389/fonc.2025.1468928)
Supplement: Supplementary file 1 [file DataSheet1.pdf]

Supplementary Material

SUPPLEMENTARY TABLE 1 Search string from the search update (July 2022).

	Term	Hits
1	Embase, MEDLINE and MEDLINE In-Process (via Ovid) search strategy	X
2	exp prostate cancer/	390,191
3	prostate.ab,ti.	508,076
4	(metasta* or advance* or malignan*).ab,ti.	4,595,061
5	(cancer or neoplas* or carcinoma* or tum$r).ab,ti.	6,109,982
6	3 and 4 and 5	150,240
7	2 or 6	425,528
8	androgen deprivation therapy/ or exp anti-androgen therapy/ or anti androgen.ab,ti. or anti-androgen.ab,ti. or androgen antagonist.ab,ti. or androgen dependent.ab,ti. or androgen-dependent.ab,ti. or androgen ablation.ab,ti. or androgen- ablation.ab,ti. or androgen blockade.ab,ti. or androgen-blockade.ab,ti. or androgen receptor.ab,ti. or androgen suppression.ab,ti. or luteinizing hormone.ab,ti. or luteinising hormone.ab,ti. or gonadotropin-releasing hormone.ab,ti. or gonadotropin releasing hormone.ab,ti. or lhrh.ab,ti. or gnrh.ab,ti. or abiraterone.ab,ti. or abiraterone acetate.ab,ti. or zytiga.ab,ti. or androgen deprivation.ab,ti. or adt.ab,ti. or docetaxel.ab,ti. or taxotere.ab,ti. or docecad.ab,ti. or docefrez.ab,ti. or zytax.ab,ti. or enzalutamide.ab,ti. or leuprolide.ab,ti. or leuprorelin.ab,ti. or lupron.ab,ti. or viadur.ab,ti. or eligard.ab,ti. or prostap.ab,ti. or buserelin.ab,ti. or seprefact.ab,ti. or cinnafact.ab,ti. or metrelef.ab,ti. or aminoglutethimide.ab,ti. or cytadren.ab,ti. or xtandi.ab,ti. or goserelin.ab,ti. or zoladex.ab,ti. or triptorelin.ab,ti. or decapeptyl.ab,ti. or diphereline.ab,ti. or gonapeptyl.ab,ti. or trelstar.ab,ti. or variopeptyl.ab,ti. or histrelin.ab,ti. or vantas.ab,ti. or supprelin.ab,ti. or degarelix.ab,ti. or firmagon.ab,ti. or antiandrogen.ab,ti. or flutamide.ab,ti. or eulexin.ab,ti. or cytomid.ab,ti. or chimax.ab,ti. or drogenil.ab,ti. or flucinom.ab,ti. or flutamin.ab,ti. or fugerel.ab,ti. or niftolide.ab,ti. or sebatrol.ab,ti. or bicalutamide.ab,ti. or casodex.ab,ti. or cosudex.ab,ti. or calutide.ab,ti. or kalumid.ab,ti. or nilutamide.ab,ti. or nilandron.ab,ti. or anandron.ab,ti. or estrogen.ab,ti. or oestrogen.ab,ti. or ketoconazole.ab,ti. or nizoral.ab,ti. or diethylstilbestrol.ab,ti. or ethinylestradiol.ab,ti. or cyproterone.ab,ti. or arn 509.ab,ti. or arn509.ab,ti. or apalutamide.ab,ti. or erleada.ab,ti. or darolutamide.ab,ti. or palbociclib.ab,ti. or ibrance.ab,ti. or ipilimumab.ab,ti. or yervoy.ab,ti.	633,483
9	exp randomized controlled trial/	1,294,945
10	randomized controlled trials as topic/	321,111
11	exp Randomization/	201,563
12	exp clinical trial/	2,661,282

13	double blind.ti,ab.	363,545
14	single blind.ti,ab.	36,651
15	(cross-over or crossover).ti,ab.	211,537
16	randomization/	201,262
17	control group/	112,260
18	(clin$ adj3 trial$).ti,ab.	1,143,692
19	randomi?ed controlled trial$.mp.	1,836,396
20	RCT.ti,ab.	75,757
21	((singl$ or doubl$ or trebl$ or tripl$) adj3 (blind$ or mask$)).mp.	606,506
22	placebo$.ti,ab.	583,407
23	(random$ adj2 allocat$).ti,ab.	91,642
24	open label.ti,ab.	149,431
25	(phase adj3 (III or "3") adj3 (study or studies or trial*)).ti,ab.	147,341
26	((equivalence or superiority or non-inferiority or noninferiority) adj3 (study or studies or trial*)).mp.	26,943
27	randomized controlled trial.pt.	573,977
28	or/9-27	4,431,405
29	7 and 8 and 28	19,673
30	limit 29 to humans	18,611
31	limit 30 to yr="2021 -Current"	2029
32	(systematic or (meta and analy*) or ((indirect or mixed) and treatment comparison)).ti.	661,865
33	(review or letter or editorial or conference abstract or conference paper or conference review).pt.	14,898,989
34	32 or 33	15,230,762
35	31 not 34	1066
36	remove duplicates from 35	710
37	CDSR and CENTRAL (via Ovid) search strategy	X
38	prostate.ab,ti.	20,350
39	(metasta* or advance* or malignan*).ab,ti.	130,855
40	(cancer or neoplas* or carcinoma* or tum$r).ab,ti.	189,971
41	38 and 39 and 40	6745
42	exp Prostatic Neoplasms/	6199
43	41 or 42	10,723
44	(anti androgen or androgen antagonist or androgen dependent or androgen ablation or androgen blockade or androgen-blockade or androgen receptor or androgen suppression or luteinizing hormone or luteinising hormone or gonadotropin-releasing hormone or gonadotropin releasing hormone or lhrh or gnrh or abiraterone or abiraterone acetate or zytiga or androgen deprivation or adt or docetaxel or taxotere or docecad or docefrez or zytax or enzalutamide or leuprolide or leuprorelin or lupron or viadur or eligard or prostap or buserelin or seprefact or cinnafact or metrelef or	34,190

	aminoglutethimide or cytadren or xtandi or goserelin or zoladex or triptorelin or decapeptyl or diphereline or gonapeptyl or trelstar or variopeptyl or histrelin or vantas or supprelin or degarelix or firmagon or antiandrogen or flutamide or eulexin or cytomid or chimax or drogenil or flucinom or flutamin or fugerel or niftolide or sebatrol or bicalutamide or casodex or cosudex or calutide or kalumid or nilutamide or nilandron or anandron or estrogen or oestrogen or ketoconazole or nizoral or diethylstilbestrol or ethinylestradiol or cyproterone or arn 509 or arn509 or apalutamide or erleada or darolutamide or palbociclib or ibrance or ipilimumab or yervoy).ab,ti.	
45	43 and 44	4884
46	limit 45 to yr="2021 -Current"	544

Databases searched: Embase, MEDLINE and MEDLINE In-Process, CDSR, and CENTRAL (all via Ovid). CDSR, Cochrane Database of Systematic Reviews.

SUPPLEMENTARY TABLE 2 AEs of interest for the eight included studies.

Study	Treatment group	N	Fatigue		Neutropenia		Hypertension		Rash		Fall		Cognitive impairment	
			Grade ≥3 AEs	Any AE	Grade ≥3 AEs	Any AE	Grade ≥3 AEs	Any AE	Grade ≥3 AEs	Any AE	Grade ≥3 AEs	Any AE	Grade ≥3 AEs	Any AE
STAMPEDE (arms G and A) (1)	AAP + ADT	948	21 (2.2)	NA	10 (1.1)*	73 (7.7)*	44 (4.6)*	299 (32)*	NA	NA	0*,†	0*,†	4 (0.42)	61 (6.4)
	Placebo + ADT	960	15 (1.6)	NA	2 (0.21)*	40 (4.2)*	13 (1.4)*	131 (14)*	NA	NA	0*,†	0*,†	2 (0.21)	36 (3.8)
STAMPEDE (arms G and C) (2)	AAP + ADT	373	8 (2.1)	NA	4 (1.1)*	NA	12 (3.2)*,†	NA	NA	NA	NA	NA	NA	NA
	Docetaxel + ADT	172	7 (4.1)	NA	22 (13)*	NA	0*,†	NA	NA	NA	NA	NA	NA	NA
STAMPEDE (arms C and A) (3)	Docetaxel + ADT	550	NA	NA	66 (12)*	NA	NA	NA	NA	NA	NA	NA	NA	NA
	Placebo + ADT	1228	NA	NA	6 (0.49)*	NA	NA	NA	NA	NA	NA	NA	NA	NA
LATITUDE; Generated from IPD; data on file(4)	AAP + ADT	597	11 (1.8)	84 (14)	4 (0.67)	9 (1.5)	125 (21)	229 (38)	0†	16 (2.7)	1 (0.17)†	11 (1.8)	0†	4 (0.7)
	Placebo + ADT	602	14 (2.3)	90 (15)	5 (0.83)	10 (1.7)	60 (10)	133 (22)	1 (0.17)†	16 (2.7)	0†	7 (1.2)	1 (0.17)†	1 (0.2)
CHAARTED (5)	Docetaxel + ADT	390	NA	NA	NA	NA	NA	NA	NA	NA	NA	NA	NA	NA
	Placebo + ADT	392	NA	NA	NA	NA	NA	NA	NA	NA	NA	NA	NA	NA
ARASENS (6)	Darolutamide + docetaxel + ADT	652	NA	216 (33)	220 (34)	256 (39)	42 (6.4)	NA	NA	NA	NA	NA	NA	NA
	Docetaxel + ADT	650	NA	214 (33)	222 (34)	252 (39)	21 (3.2)	NA	NA	NA	NA	NA	NA	NA
ARCHES (7)	Enzalutamide + ADT	572	16 (2.8)‡	184 (32)	4 (0.70) ‡	8 (1.4)	29 (5.1) ‡	82 (14)	0†, ‡	22 (3.8)	7 (1.2) ‡	58 (10)	4 (0.70)†, ‡	38 (6.6)
	Placebo + ADT	574	11 (1.9) ‡	118 (21)	2 (0.35) ‡	4 (0.70)	13 (2.3) ‡	39 (6.8)	0†,‡	10 (1.7)	3 (0.52) ‡	19 (3.3)	0†,‡	15 (2.6)
GETUG-AFU 15 (8)	Docetaxel + ADT	189	13 (6.9)	140 (74)	61 (32)†	94 (50)	NA	NA	NA	NA	NA	NA	NA	NA
	Placebo + ADT	186	2 (1.1)	37 (20)	0†	5 (2.7)	NA	NA	NA	NA	NA	NA	NA	NA

TITAN; Generated from IPD; data on file	APA + ADT	524	8 (1.5)	107 (20)	4 (0.8)	16 (3.1)	54 (10)	102 (19)	19 (3.6)	106 (20)	7 (1.3)	49 (9.4)	2 (0.38)[†]	6 (1.1)
	Placebo + ADT	527	7 (1.3)	89 (17)	1 (0.2)	15 (2.8)	47 (8.9)	84 (16)	3 (0.57)	23 (4.4)	5 (0.95)	37 (7.0)	0[†]	2 (0.38)
PEACE-1 (9)	AAP + docetaxel + ADT (±RT)	347	10 (2.9)	NA	34 (9.8)[†]	NA	76 (22)	NA	NA	NA	NA	NA	NA	NA
	Docetaxel + ADT (±RT)	350	15 (4.3)	NA	32 (9.1)[†]	NA	45 (13)	NA	NA	NA	NA	NA	NA	NA
	AAP + ADT (±RT)	226	3 (1.3)	NA	0[†]	NA	66 (29)	NA	NA	NA	NA	NA	NA	NA
	Placebo + ADT (±RT)	237	0	NA	0[†]	NA	38 (16)	NA	NA	NA	NA	NA	NA	NA

Data are presented as n (%). AAP, abiraterone acetate plus prednisone; ADT, androgen-deprivation therapy; AE, adverse event; IPD, individual participant data; NA, not available; RT, radiotherapy.

*Safety data for M1 + M0 STAMPEDE population were reported together.

[†]A continuity correction was performed where n=0.5 was added to each AE when performing the NMAs.

[‡]Grade 3–4 AEs instead of grade 3–5 were included based on availability.

SUPPLEMENTARY FIGURE 1

Network plots for (**A**) grade ≥3 adverse events (AEs) and any AE, and (**B**) serious AEs (SAEs). AAP, abiraterone acetate plus prednisone; ADT, androgen-deprivation therapy.

(A) Grade ≥3 AEs and any AE

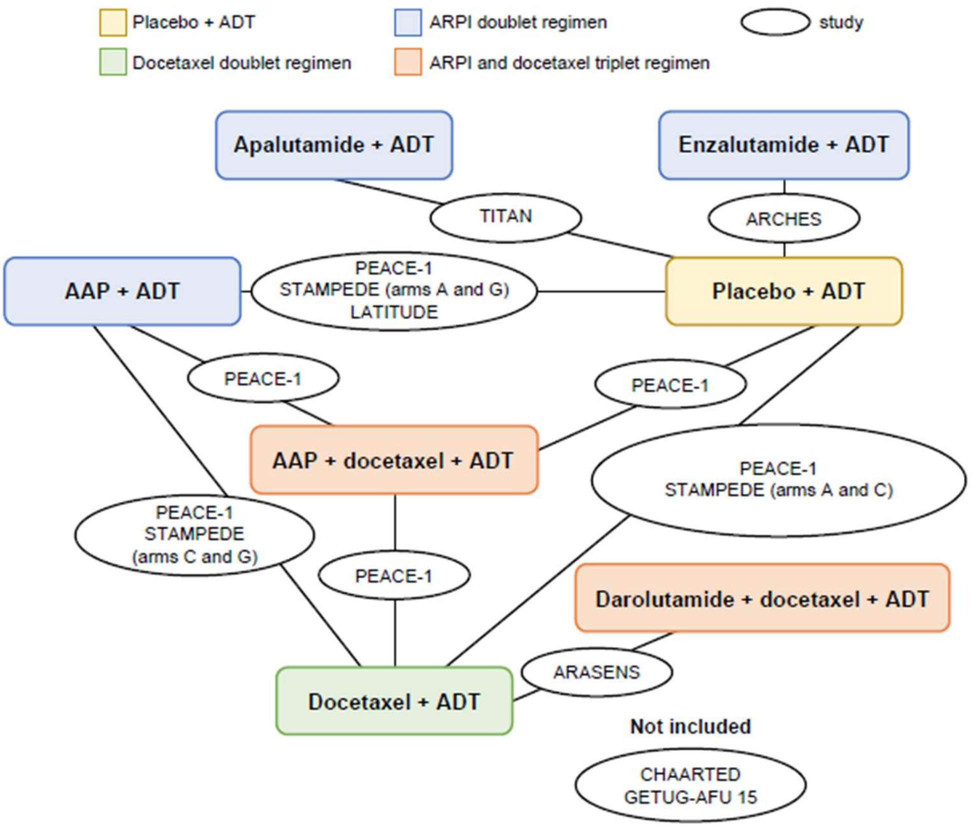

(B) SAEs

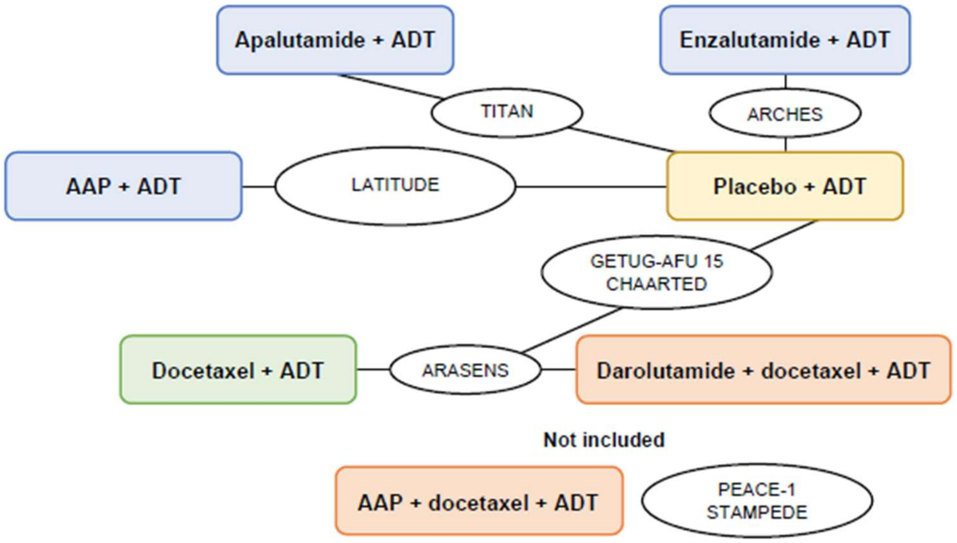

SUPPLEMENTARY FIGURE 2

Random-effects model for Bayesian network meta-analysis comparison results for (**A**) grade ≥3 adverse events (AEs), (**B**) serious AEs (SAEs), and (**C**) any AEs. Data are RR (95% Crl) and P (RR<1). AAP, abiraterone acetate plus prednisone; ADT, androgen-deprivation therapy; ARPI, androgen receptor pathway inhibitor; CrI, credible interval; P, probability; RR, relative risk.

(A) Grade ≥3 AEs

Placebo + ADT; Docetaxel doublet regimen; ARPI doublet regimen; ARPI and docetaxel triplet regimen

RR (95% Crl) P (RR<1)	Placebo + ADT	Apalutamide + ADT	Enzalutamide + ADT	Docetaxel + ADT	Darolutamide + docetaxel + ADT	AAP + ADT	AAP + docetaxel + ADT
Placebo + ADT		0.85 (0.58–1.48) 80	0.75 (0.53–1.24) 92	0.70 (0.57–0.93) 99	0.66 (0.48–1.15) 95	0.66 (0.55–0.83) 100	0.63 (0.49–0.93) 98
Apalutamide + ADT	1.18 (0.68–1.74) 20		0.88 (0.47–1.65) 71	0.83 (0.46–1.33) 83	0.78 (0.42–1.52) 83	0.78 (0.44–1.21) 90	0.75 (0.41–1.28) 90
Enzalutamide + ADT	1.34 (0.81–1.88) 8	1.13 (0.61–2.15) 29		0.94 (0.55–1.46) 64	0.89 (0.49–1.68) 70	0.89 (0.52–1.32) 76	0.85 (0.49–1.41) 80
Docetaxel + ADT	1.42 (1.07–1.76) 1	1.21 (0.75–2.17) 17	1.06 (0.69–1.83) 36		0.94 (0.70–1.49) 66	0.94 (0.73–1.18) 74	0.90 (0.66–1.29) 79
Darolutamide + docetaxel + ADT	1.50 (0.87–2.09) 5	1.27 (0.66–2.41) 17	1.12 (0.59–2.04) 30	1.06 (0.67–1.43) 34		1.00 (0.59–1.41) 50	0.95 (0.55–1.49) 61
AAP + ADT	1.51 (1.21–1.81) 0	1.28 (0.83–2.28) 10	1.13 (0.76–1.93) 24	1.06 (0.85–1.38) 26	1.00 (0.71–1.71) 50		0.95 (0.73–1.38) 65
AAP + docetaxel + ADT	1.58 (1.07–2.03) 2	1.34 (0.78–2.44) 10	1.18 (0.71–2.06) 20	1.11 (0.77–1.51) 21	1.05 (0.67–1.81) 39	1.05 (0.72–1.38) 35	

(B) SAEs

RR (95% Crl) P (RR<1)	Placebo + ADT	Apalutamide + ADT	AAP + ADT	Enzalutamide + ADT	Docetaxel + ADT	Darolutamide + docetaxel + ADT
Placebo + ADT		0.79 (0.35–2.81) 69	0.75 (0.34–2.65) 73	0.65 (0.32–2.16) 81	0.26 (0.22–0.31) 100	0.25 (0.22–0.37) 100
Apalutamide + ADT	1.26 (0.36–2.85) 31		0.95 (0.21–4.22) 53	0.82 (0.19–3.50) 63	0.33 (0.09–0.74) 100	0.33 (0.09–0.78) 99
AAP + ADT	1.33 (0.38–2.93) 27	1.05 (0.24–4.69) 47		0.87 (0.20–3.62) 60	0.34 (0.10–0.76) 100	0.34 (0.10–0.80) 99
Enzalutamide + ADT	1.54 (0.46–3.14) 19	1.22 (0.29–5.21) 37	1.15 (0.28–4.90) 40		0.40 (0.12–0.81) 100	0.40 (0.12–0.86) 99
Docetaxel + ADT	3.91 (3.20–4.49) 0	3.07 (1.36–11.04) 0	2.91 (1.31–10.42) 0	2.51 (1.24–8.44) 0		0.99 (0.88–1.35) 57
Darolutamide + docetaxel + ADT	3.93 (2.67–4.57) 0	3.06 (1.28–10.98) 1	2.90 (1.25–10.38) 1	2.50 (1.17–8.41) 1	1.01 (0.74–1.13) 43	

(C) Any AE

RR (95% Crl) P (RR<1)	Placebo + ADT	Apalutamide + ADT	Enzalutamide + ADT	AAP + ADT	AAP + docetaxel + ADT	Docetaxel + ADT	Darolutamide + docetaxel + ADT
Placebo + ADT		1.00 (0.97–1.09) 55	0.99 (0.97–1.05) 78	0.98 (0.97–1.00) 97	0.97 (0.96–1.02) 93	0.97 (0.96–0.99) 100	0.97 (0.95–0.98) 100
Apalutamide + ADT	1.00 (0.92–1.03) 45		0.99 (0.91–1.06) 65	0.99 (0.90–1.02) 82	0.98 (0.89–1.03) 89	0.97 (0.89–1.00) 98	0.97 (0.89–1.00) 99
Enzalutamide + ADT	1.01 (0.95–1.03) 22	1.01 (0.95–1.10) 35		0.99 (0.93–1.02) 73	0.98 (0.93–1.03) 86	0.98 (0.92–1.00) 97	0.98 (0.92–1.00) 98
AAP + ADT	1.02 (1.00–1.04) 3	1.01 (0.98–1.11) 18	1.01 (0.98–1.07) 27		0.99 (0.97–1.04) 79	0.99 (0.97–1.00) 96	0.98 (0.97–1.00) 98
AAP + docetaxel + ADT	1.03 (0.98–1.05) 7	1.03 (0.97–1.12) 11	1.02 (0.97–1.08) 14	1.01 (0.97–1.03) 21		1.00 (0.95–1.01) 62	1.00 (0.95–1.01) 78
Docetaxel + ADT	1.03 (1.02–1.05) 0	1.03 (1.00–1.12) 2	1.02 (1.00–1.09) 3	1.01 (1.00–1.03) 4	1.00 (0.99–1.05) 38		1.00 (0.99–1.01) 86
Darolutamide + docetaxel + ADT	1.03 (1.02–1.05) 0	1.03 (1.00–1.13) 1	1.02 (1.00–1.09) 2	1.02 (1.00–1.04) 2	1.00 (0.99–1.05) 22	1.00 (0.99–1.01) 14	

SUPPLEMENTARY FIGURE 3

Random-effects model results for relative risk (RR) of aggregated safety outcomes following systemic therapies versus androgen-deprivation therapy (ADT) alone. (A) grade ≥3 adverse events (AEs), (B) serious AEs (SAEs), and (C) any AE. All data are rounded from random-effects model. AAP, abiraterone acetate plus prednisone; CrI, credible interval.

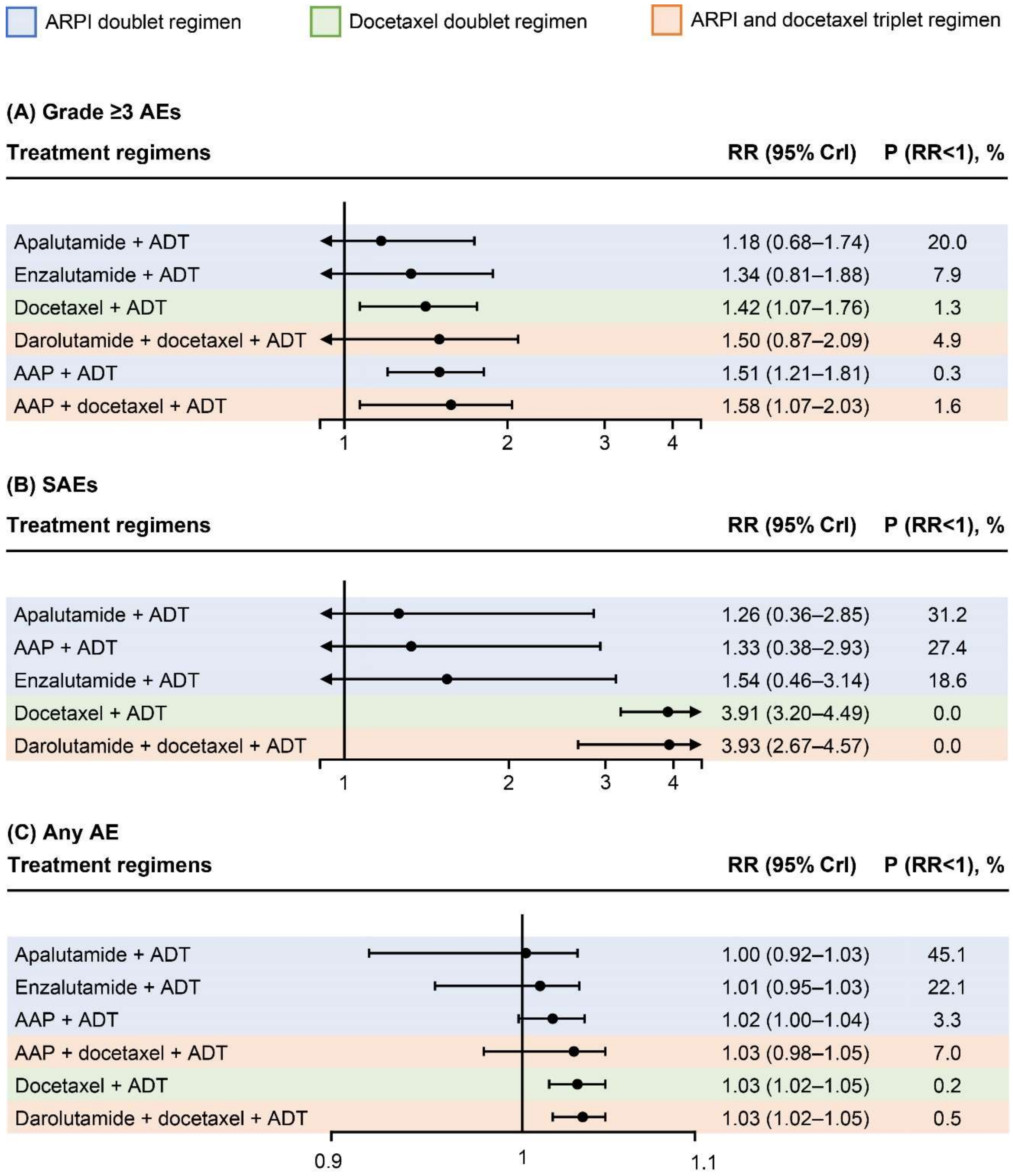

SUPPLEMENTARY FIGURE 4

Relative risk (RR) for adverse events (AEs) of interest following systemic therapies versus androgen-deprivation therapy (ADT). (**A**) fatigue, (**B**) neutropenia, (**C**) hypertension, (**D**) rash, (**E**) fall, and (**F**) cognitive impairment. All data are rounded from fixed-effects model. AAP, abiraterone acetate plus prednisone; ARPI, androgen receptor pathway inhibitor; CrI, credible interval.

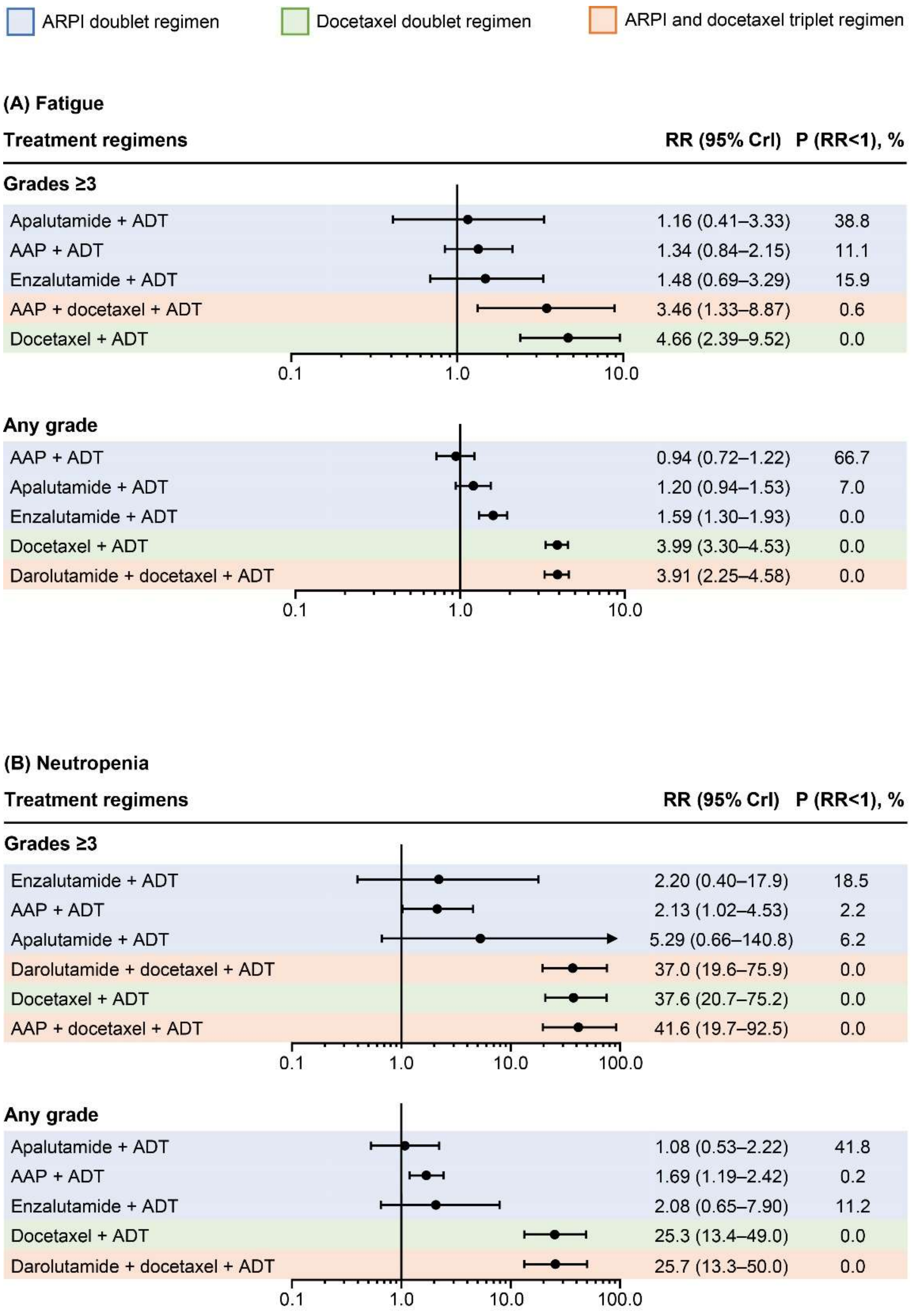

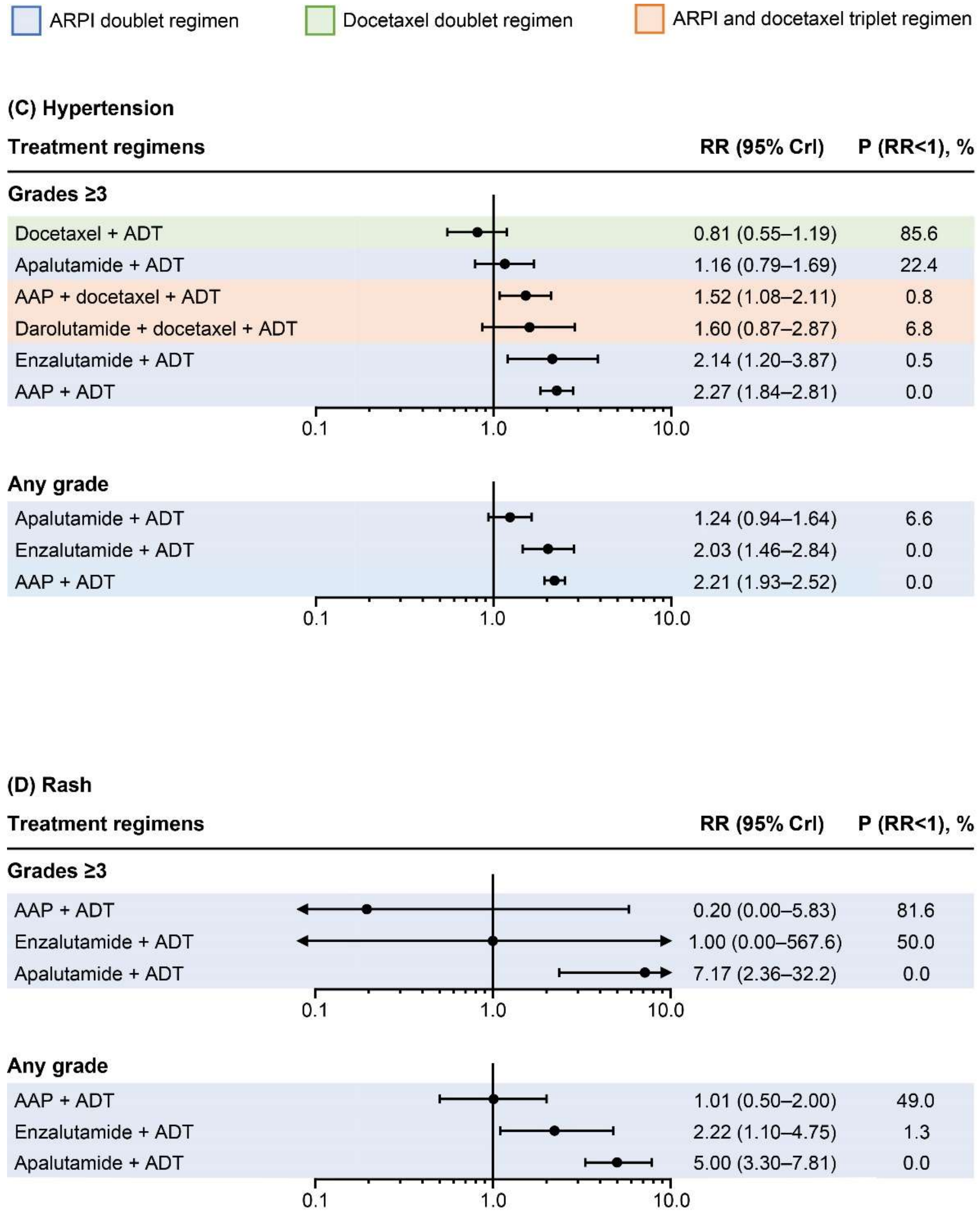
ARPI doublet regimen
Docetaxel doublet regimen
ARPI and docetaxel triplet regimen
(C) Hypertension
Treatment regimens
RR (95% CrI)
P (RR<1), %
Grades ≥3
Docetaxel + ADT
0.81 (0.55–1.19)
85.6
Apalutamide + ADT
1.16 (0.79–1.69)
22.4
AAP + docetaxel + ADT
1.52 (1.08–2.11)
0.8
Darolutamide + docetaxel + ADT
1.60 (0.87–2.87)
6.8
Enzalutamide + ADT
2.14 (1.20–3.87)
0.5
AAP + ADT
2.27 (1.84–2.81)
0.0
0.1
1.0
10.0
Any grade
Apalutamide + ADT
1.24 (0.94–1.64)
6.6
Enzalutamide + ADT
2.03 (1.46–2.84)
0.0
AAP + ADT
2.21 (1.93–2.52)
0.0
0.1
1.0
10.0
(D) Rash
Treatment regimens
RR (95% CrI)
P (RR<1), %
Grades ≥3
AAP + ADT
0.20 (0.00–5.83)
81.6
Enzalutamide + ADT
1.00 (0.00–567.6)
50.0
Apalutamide + ADT
7.17 (2.36–32.2)
0.0
0.1
1.0
10.0
Any grade
AAP + ADT
1.01 (0.50–2.00)
49.0
Enzalutamide + ADT
2.22 (1.10–4.75)
1.3
Apalutamide + ADT
5.00 (3.30–7.81)
0.0
0.1
1.0
10.0

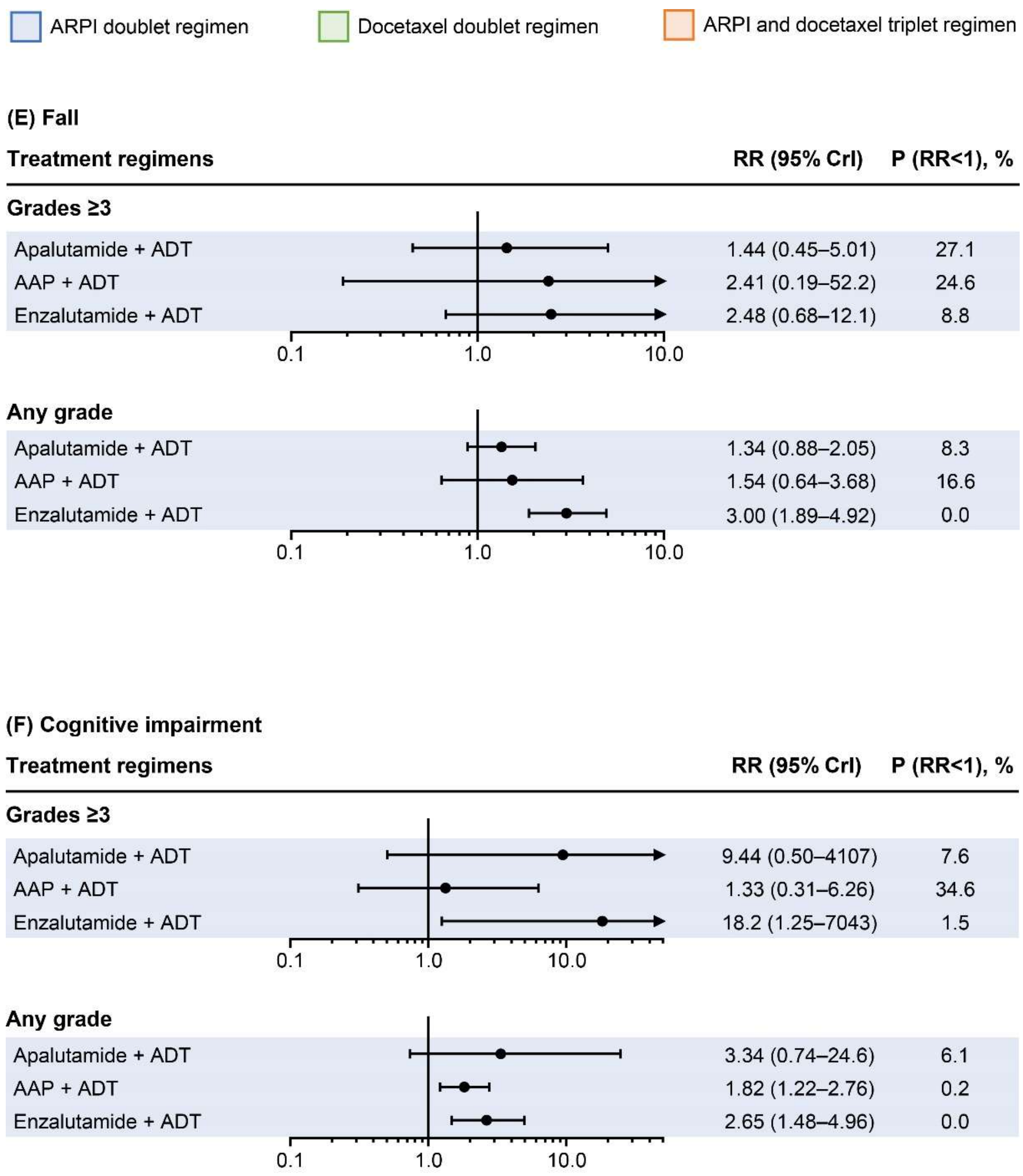
ARPI doublet regimen
Docetaxel doublet regimen
ARPI and docetaxel triplet regimen
(E) Fall
Treatment regimens
RR (95% CrI)
P (RR<1), %
Grades ≥3
Apalutamide + ADT
1.44 (0.45–5.01)
27.1
AAP + ADT
2.41 (0.19–52.2)
24.6
Enzalutamide + ADT
2.48 (0.68–12.1)
8.8
0.1
1.0
10.0
Any grade
Apalutamide + ADT
1.34 (0.88–2.05)
8.3
AAP + ADT
1.54 (0.64–3.68)
16.6
Enzalutamide + ADT
3.00 (1.89–4.92)
0.0
0.1
1.0
10.0
(F) Cognitive impairment
Treatment regimens
RR (95% CrI)
P (RR<1), %
Grades ≥3
Apalutamide + ADT
9.44 (0.50–4107)
7.6
AAP + ADT
1.33 (0.31–6.26)
34.6
Enzalutamide + ADT
18.2 (1.25–7043)
1.5
0.1
1.0
10.0
Any grade
Apalutamide + ADT
3.34 (0.74–24.6)
6.1
AAP + ADT
1.82 (1.22–2.76)
0.2
Enzalutamide + ADT
2.65 (1.48–4.96)
0.0
0.1
1.0
10.0

SUPPLEMENTARY FIGURE 5

Sensitivity analysis: relative risk (RR) for aggregated safety outcomes following systemic therapies versus androgen-deprivation therapy (ADT) alone with ENZAMET included. (**A**) grade ≥3 adverse events (AEs), (**B**) serious AEs (SAEs), and (**C**) any AE. All data are rounded from fixed-effects model. ARPI, androgen receptor pathway inhibitor; CrI, credible interval.

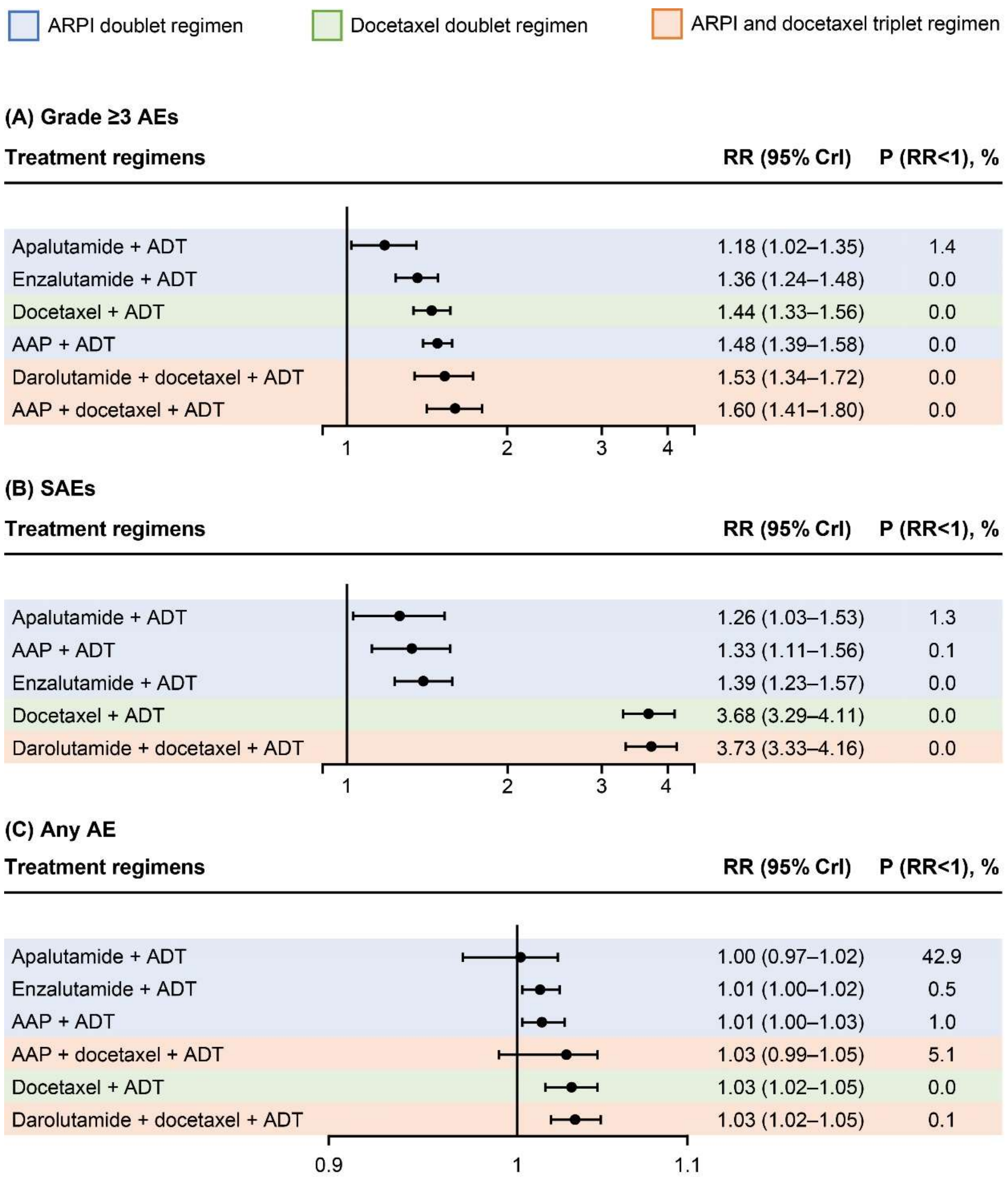

SUPPLEMENTARY FIGURE 6

Sensitivity analysis: relative risk (RR) for aggregated safety outcomes following systemic therapies versus ADT alone without STAMPEDE included. (**A**) grade ≥3 AEs and (**B**) any AE. Because STAMPEDE did not report SAEs, the sensitivity analysis for this endpoint was not performed as it would encompass the exact same trials as in the base-case analysis. All data are rounded from fixed-effects model. AAP, abiraterone acetate plus prednisone; ADT, androgen-deprivation therapy; AE, adverse event; ARPI, androgen receptor pathway inhibitor; CrI, credible interval; RR, relative risk; SAE, serious AE.

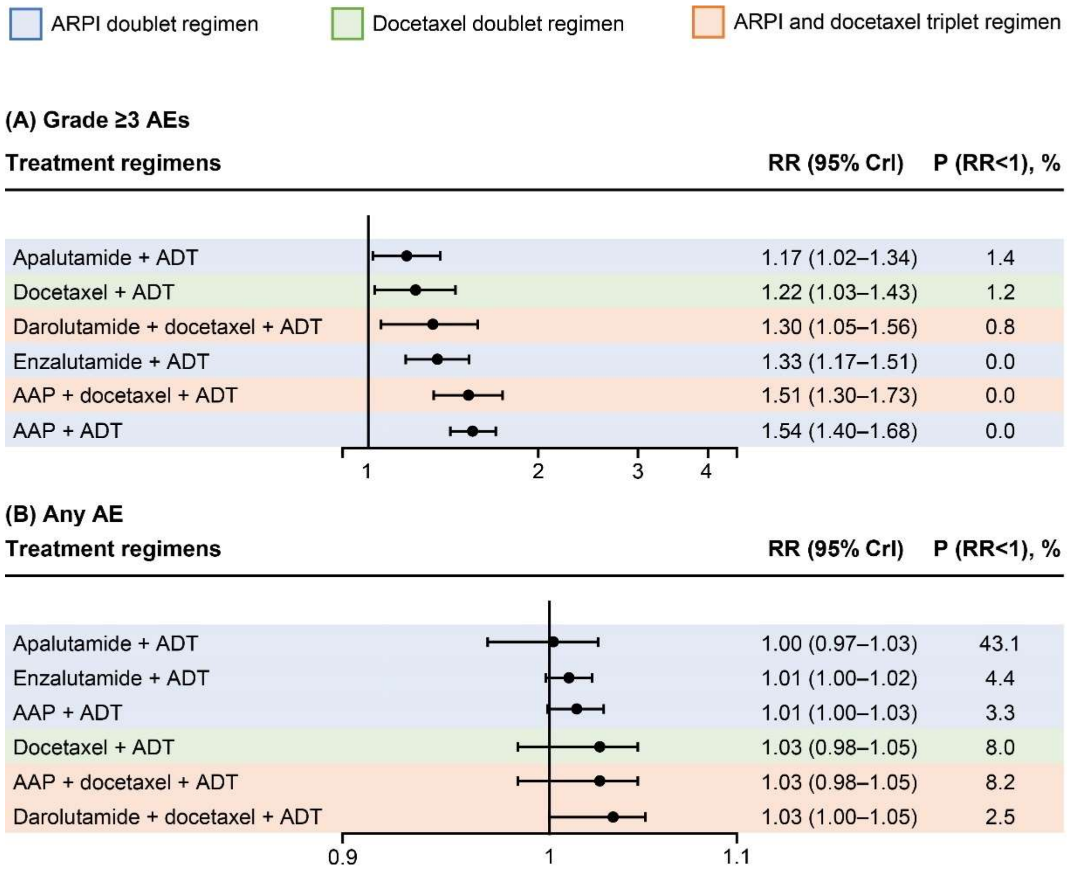

SUPPLEMENTARY FIGURE 7

Sensitivity analysis: relative risk (RR) for aggregated safety outcomes following systemic therapies versus androgen-deprivation therapy (ADT) alone without LATITUDE included. (**A**) grade ≥3 adverse events (AEs), (**B**) serious AEs (SAEs), and (**C**) any AE. All data are rounded from fixed-effects model. AAP, abiraterone acetate plus prednisone; ADT, androgen-deprivation therapy; ARPI, androgen receptor pathway inhibitor; CrI, credible interval.

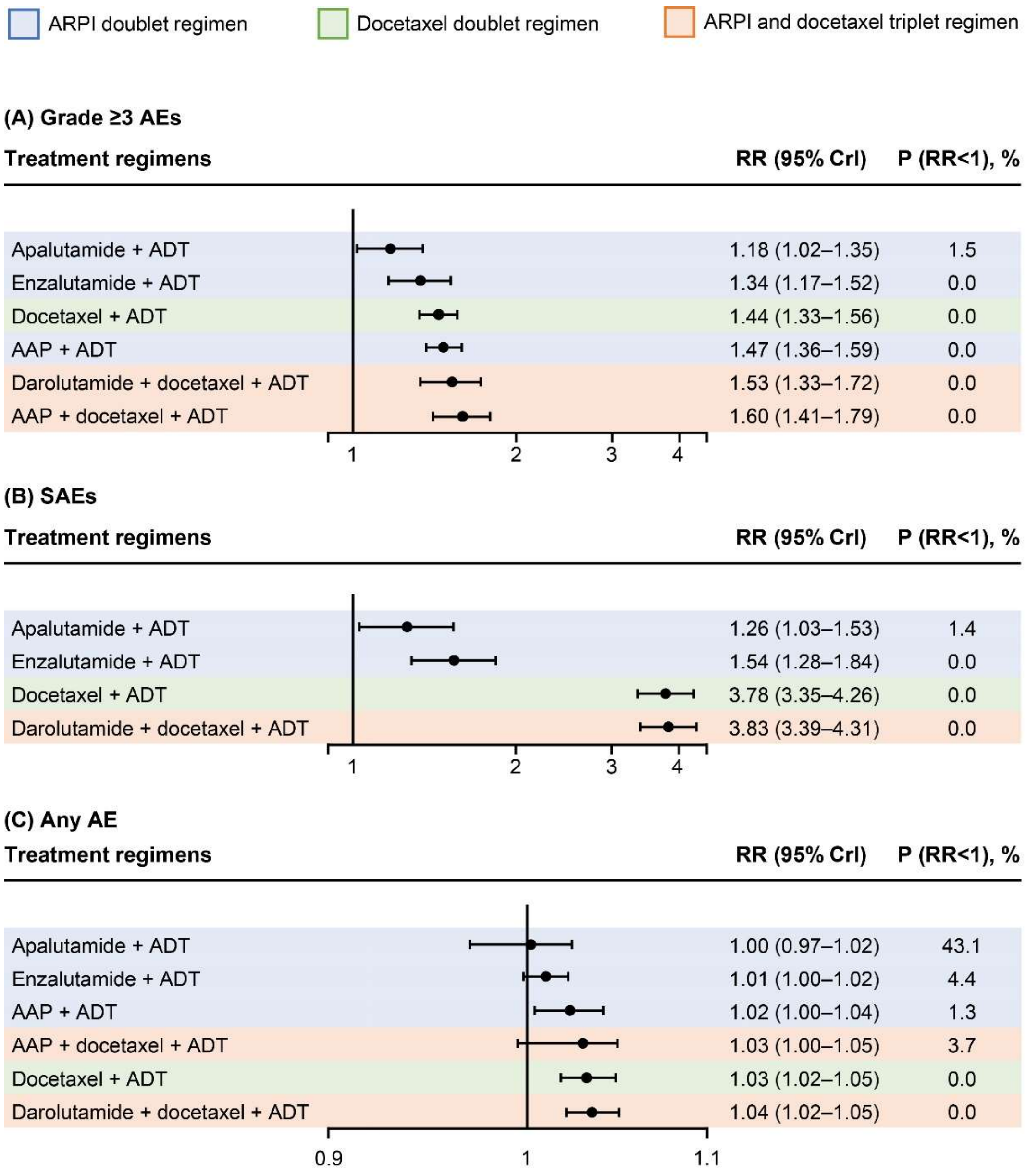

SUPPLEMENTARY FIGURE 8

Sensitivity analysis: relative risk (RR) for aggregated safety outcomes following systemic therapies versus androgen-deprivation therapy (ADT) alone without PEACE-1 included. (**A**) grade ≥3 adverse events (AEs) and (**B**) any AE. Because PEACE-1 did not report SAEs, the sensitivity analysis for this endpoint was not performed as it would encompass the exact same trials as in the base-case analysis. All data are rounded from fixed-effects model. AAP, abiraterone acetate plus prednisone; ARPI, androgen receptor pathway inhibitor; CrI, credible interval.

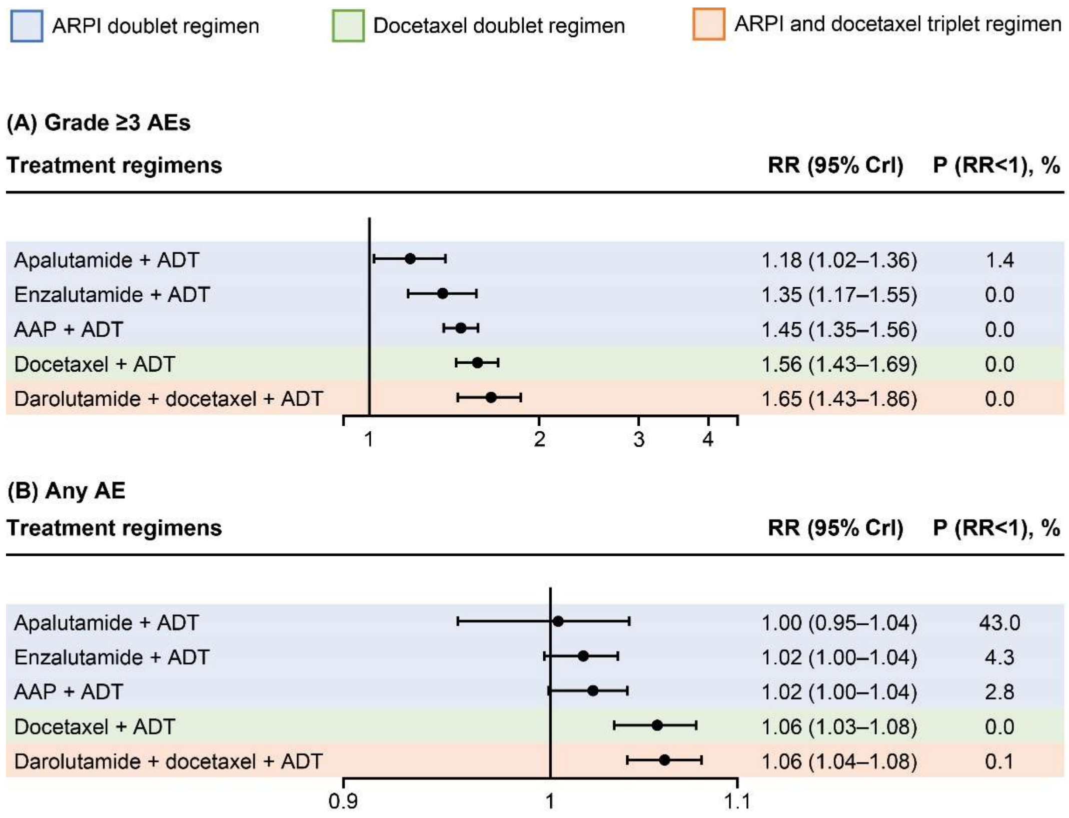

SUPPLEMENTARY FIGURE 9

Sensitivity analysis: relative risk (RR) for aggregated safety outcomes following systemic therapies versus androgen-deprivation therapy (ADT) alone without ARCHES included for grade ≥3 adverse events (AEs). All data are rounded from fixed-effects model. AAP, abiraterone acetate plus prednisone; ARPI, androgen receptor pathway inhibitor; CrI, credible interval.

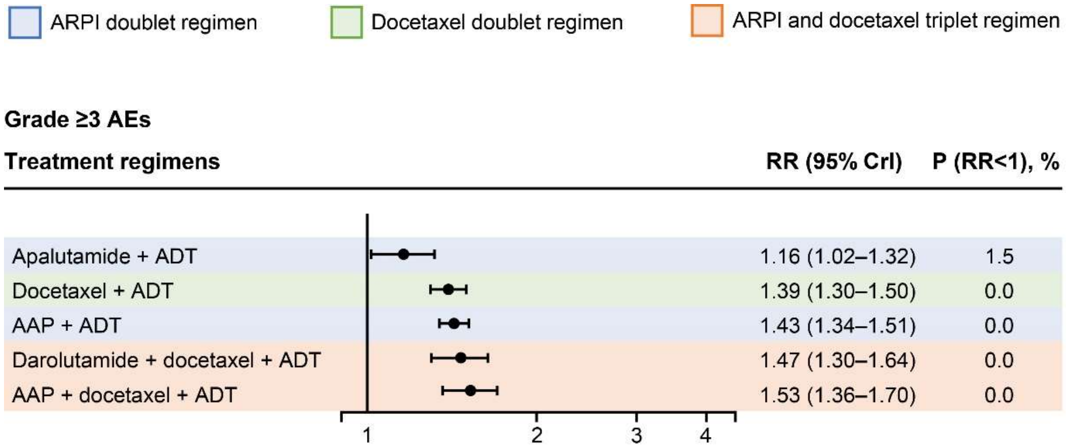

SUPPLEMENTARY FIGURE 10

Sensitivity analysis: relative risk (RR) for aggregated safety outcomes following systemic therapies versus androgen-deprivation therapy (ADT) alone where patients from TITAN who received prior docetaxel to study treatment were not included. (**A**) grade ≥3 adverse events (AEs), (**B**) serious AEs (SAEs), and (**C**) any AE. All data are rounded from fixed-effects model. AAP, abiraterone acetate plus prednisone; ARPI, androgen receptor pathway inhibitor; CrI, credible interval.

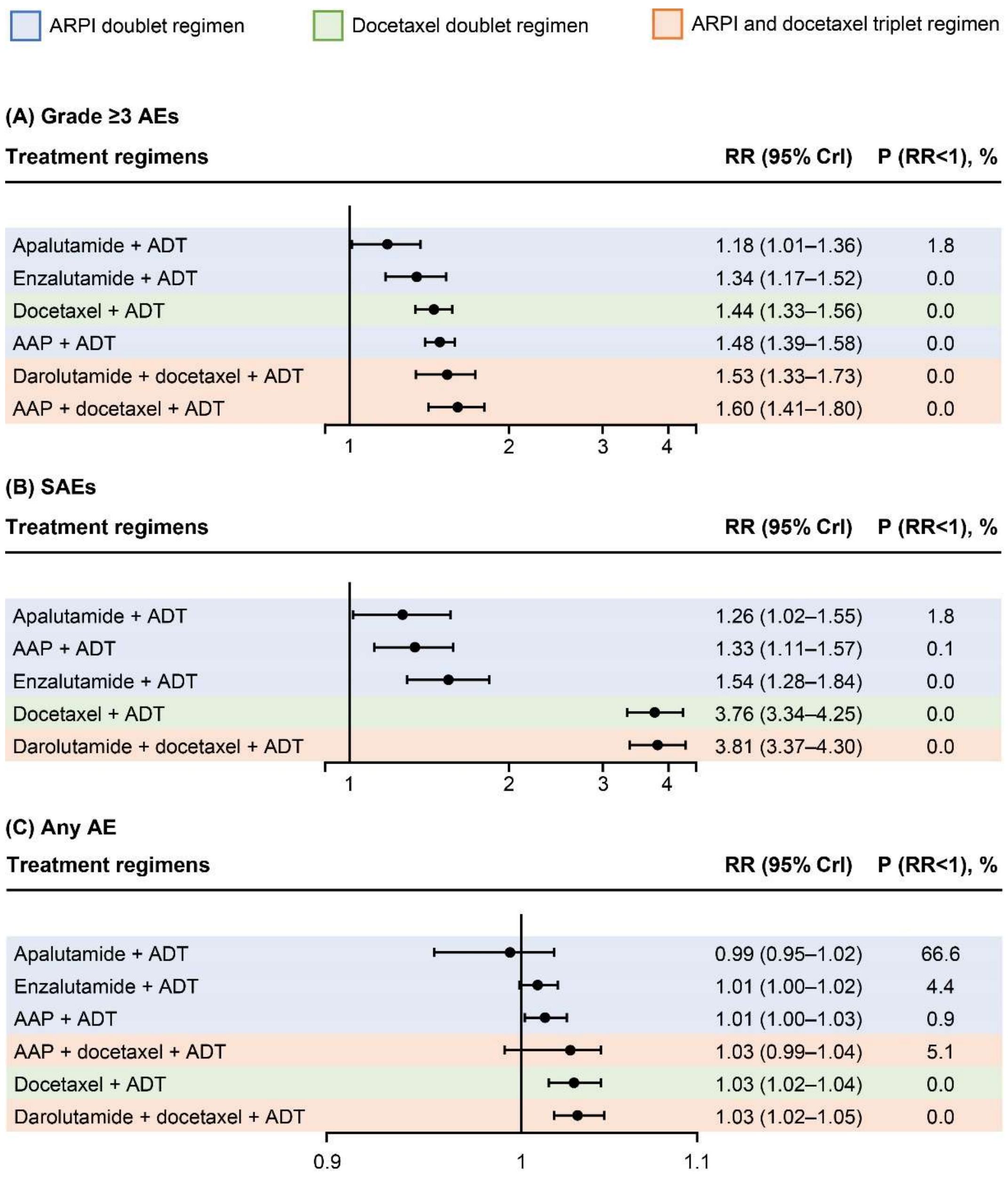

SUPPLEMENTARY FIGURE 11

Sensitivity analysis: relative risk (RR) for aggregated safety outcomes following systemic therapies versus androgen-deprivation therapy (ADT) alone including only licensed treatment regimens for metastatic hormone-sensitive prostate cancer in Europe. (**A**) Grade ≥3 adverse events (AEs) and (**B**) any AE are shown using data from TITAN, ARCHES, LATITUDE, STAMPEDE arms A and C, and ARASENS only. PEACE-1 was not included. Because STAMPEDE did not report SAEs, the sensitivity analysis for this endpoint was not performed as it would encompass the exact same trials as in the base-case analysis. All data are rounded from fixed-effects model. AAP, abiraterone acetate plus prednisone; ARPI, androgen receptor pathway inhibitor; CrI, credible interval.

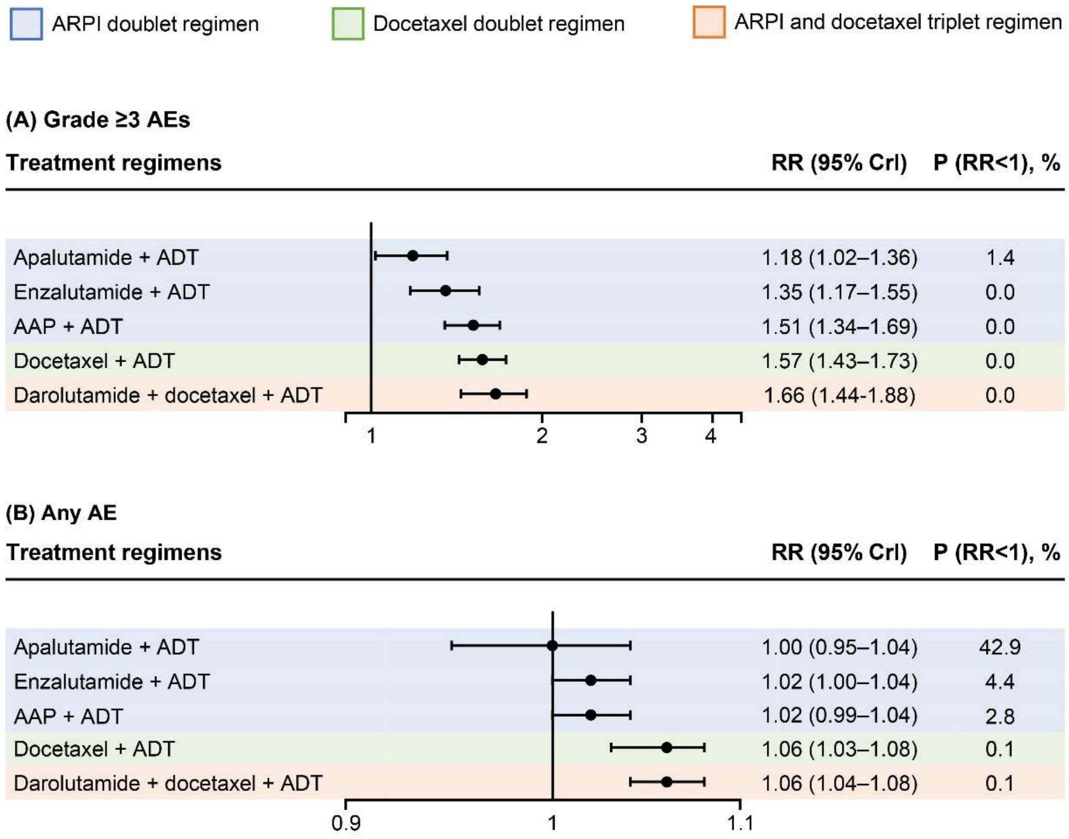

References

1. James ND, de Bono JS, Spears MR, Clarke NW, Mason MD, Dearnaley DP, et al. Abiraterone for prostate cancer not previously treated with hormone therapy. N Engl J Med. (2017) 377(4):338-51. 10.1056/NEJMoa1702900
2. Sydes MR, Spears MR, Mason MD, Clarke NW, Dearnaley DP, de Bono JS, et al. Adding abiraterone or docetaxel to long-term hormone therapy for prostate cancer: directly randomised data from the STAMPEDE multi-arm, multi-stage platform protocol. Ann Oncol. (2018) 29(5):1235-48. 10.1093/annonc/mdy072
3. James ND, Sydes MR, Clarke NW, Mason MD, Dearnaley DP, Spears MR, et al. Addition of docetaxel, zoledronic acid, or both to first-line long-term hormone therapy in prostate cancer (STAMPEDE): survival results from an adaptive, multiarm, multistage, platform randomised controlled trial. Lancet. (2016) 387(10024):1163-77. 10.1016/S0140-6736(15)01037-5
4. Fizazi K, Tran N, Fein L, Matsubara N, Rodriguez-Antolin A, Alekseev BY, et al. Abiraterone acetate plus prednisone in patients with newly diagnosed high-risk metastatic castration-sensitive prostate cancer (LATITUDE): final overall survival analysis of a randomised, double-blind, phase 3 trial. Lancet Oncol. (2019) 20(5):686-700. 10.1016/S1470-2045(19)30082-8
5. ClinicalTrials.gov. Androgen ablation therapy with or without chemotherapy in treating patients with metastatic prostate cancer (CHAARTED). https://clinicaltrials.gov/study/NCT00309985 [Accessed August 6, 2025]
6. Smith MR, Hussain M, Saad F, Fizazi K, Sternberg CN, Crawford ED, et al. Darolutamide and survival in metastatic, hormone-sensitive prostate cancer. N Engl J Med. (2022) 386(12):1132-42. 10.1056/NEJMoa2119115
7. Armstrong AJ, Azad AA, Iguchi T, Szmulewitz RZ, Petrylak DP, Holzbeierlein J, et al. Improved survival with enzalutamide in patients with metastatic hormone-sensitive prostate cancer. J Clin Oncol. (2022) 40(15):1616-22. 10.1200/JCO.22.00193
8. Gravis G, Fizazi K, Joly F, Oudard S, Priou F, Esterni B, et al. Androgen-deprivation therapy alone or with docetaxel in non-castrate metastatic prostate cancer (GETUG-AFU 15): a randomised, open-label, phase 3 trial. Lancet Oncol. (2013) 14(2):149-58. 10.1016/S1470-2045(12)70560-0
9. Fizazi K, Foulon S, Carles J, Roubaud G, McDermott R, Flechon A, et al. Abiraterone plus prednisone added to androgen deprivation therapy and docetaxel in de novo metastatic castration-sensitive prostate cancer (PEACE-1): a multicentre, open-label, randomised, phase 3 study with a 2 x 2 factorial design. Lancet. (2022) 399(10336):1695-707. 10.1016/S0140-6736(22)00367-1
